# Three new subfamilies of skipper butterflies (Lepidoptera, Hesperiidae)

**DOI:** 10.3897/zookeys.861.34686

**Published:** 2019-07-08

**Authors:** Jing Zhang, Qian Cong, Jinhui Shen, Ernst Brockmann, Nick V. Grishin

**Affiliations:** 1 Departments of Biophysics and Biochemistry, University of Texas Southwestern Medical Center, 5323 Harry Hines Blvd, Dallas, TX, 75390-9050, USA University of Texas Southwestern Medical Center Dallas United States of America; 2 Institute for Protein Design and Department of Biochemistry, University of Washington, 1959 NE Pacific Street, HSB J-405, Seattle, WA, 98195, USA University of Washington Seattle United States of America; 3 Laubacher Str. 4, 35423 Lich, Hessen, Germany Unaffiliated Seattle United States of America; 4 Howard Hughes Medical Institute, Chevy Chase, MD 20815-6789, USA Howard Hughes Medical Institute Chevy Chase United States of America

**Keywords:** Africa, Asia, genomics, higher classification, phylogeny

## Abstract

We obtained and analyzed whole genome data for more than 160 representatives of skipper butterflies (family Hesperiidae) from all known subfamilies, tribes and most distinctive genera. We found that two genera, *Katreus* Watson, 1893 and *Ortholexis* Karsch, 1895, which are sisters, are well-separated from all other major phylogenetic lineages and originate near the base of the Hesperiidae tree, prior to the origin of some subfamilies. Due to this ancient origin compared to other subfamilies, this group is described as Katreinae Grishin, **subfam. n.** DNA sequencing of primary type specimens reveals that *Ortholexismelichroptera* Karsch, 1895 is not a female of *Ortholexisholocausta* Mabille, 1891, but instead a female of *Ortholexisdimidia* Holland, 1896. This finding establishes *O.dimidia* as a junior subjective synonym of *O.melichroptera*. Furthermore, we see that *Chamunda* Evans, 1949 does not originate within Pyrginae Burmeister, 1878, but, unexpectedly, forms an ancient lineage of its own at the subfamily rank: Chamundinae Grishin, **subfam. n**. Finally, a group of two sister genera, *Barca* de Nicéville, 1902 and *Apostictopterus* Leech, [1893], originates around the time Hesperiinae Latreille, 1809 have split from their sister clade. A new subfamily Barcinae Grishin, **subfam. n.** sets them apart from all other Hesperiidae.

## Introduction

**Figure 1.**
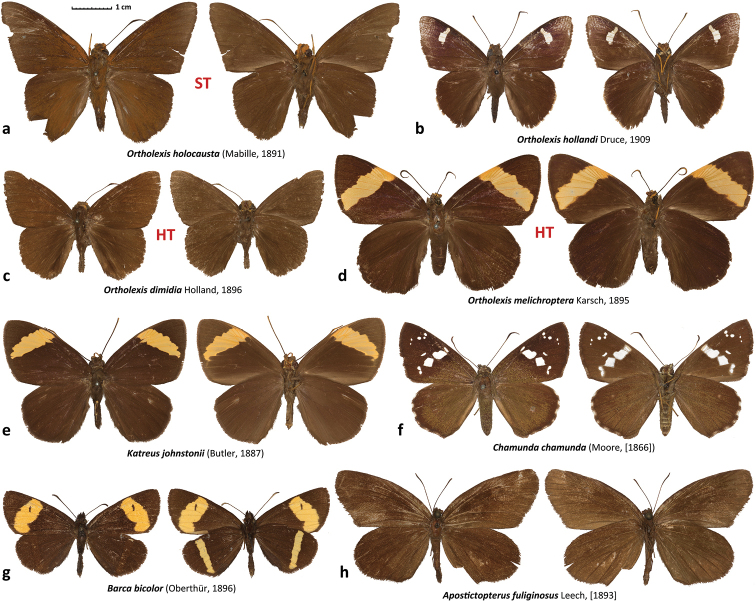
Sequenced specimens from the new Hesperiidae subfamilies. DNA sample numbers are given for each specimen, additional data are in the Suppl. material 1: Table S1 **a***Ortholexisholocausta* syntype, NVG-18053C02 **b***Ortholexishollandi*, NVG-18082A08 **c***Ortholexismelichroptera*, holotype of *Acallopistesdimidia* Holland, 1896; NVG-18053C05 **d***Ortholexismelichroptera*, holotype, NVG-18053A06 **e***Katreusjohnstonii*, NVG-18053B05 **f***Chamundachamunda*, NVG-18086E02 **g***Barcabicolor*, NVG-17069C10 **h***Apostictopterusfuliginosus*, NVG-17069C12.

New methods bring new discoveries. While careful expert-driven morphological analysis can be insightful in revealing synapomorphies and predicting evolutionary relationships between animals, DNA sequences offer additional insights. Phylogenetic analysis at the genomic scale is expected to give an unprecedented resolution and clarify many questions, providing a firm basis for the best taxonomic classification. Butterflies are attracting attention with a number of large scale phylogeny studies published recently (Espeland et al. 2015; Cao et al. 2016; Espeland et al. 2018; Seraphim et al. 2018; Toussaint et al. 2018; Li et al. 2019;). The butterfly family Hesperiidae (skippers), which includes butterflies with stout bodies, large heads and rapid wing beats, is still comparatively less known. Groundbreaking DNA analysis by Warren et al. (2008, 2009) based on several genes revealed many new phylogenetic relationships compared to the last comprehensive morphological treatment (Evans 1937, 1949, 1951, 1952, 1953, 1955) and offered an updated classification of Hesperiidae. Additional insights came from follow-up studies (Sahoo et al. 2016; Sahoo et al. 2017; Espeland et al. 2018; Toussaint et al. 2018; Li et al. 2019; Zhang et al. 2019) posing questions about phylogenetic and taxonomic placement of genera such as *Ortholexis* Karsch, 1895, *Barca* de Nicéville, 1902 and *Apostictopterus* Leech, 1893 (Fig. 1).

Here, we tackle the questions about deep phylogeny of Hesperiidae using whole genome shotgun analysis. We selected 160 representative species of skippers that cover all known subfamilies and tribes, including some genera that we thought would be interesting to analyze at the genomic scale. To make this work taxonomically sound, we used type genera and their type species where possible, and for some species used their primary type specimens. Our genomic methods break the time barrier and allow us to work with specimens more than a century old from museum collections. We find that while the backbone of the current classification of Hesperiidae stands the test of genomic data (Li et al. 2019), unexpected deep divergence of some groups awards them the status of subfamilies that are described here.

## Materials and methods

Bodies of freshly collected specimens were stored in RNAlater, and their wings and genitalia dried and kept in envelopes to address possible misidentification issues. DNA was extracted from a piece of tissue of these specimens. For specimens in museum collections, DNA was extracted either from the abdomen or from a leg. The abdomen was gently pushed from above and below (while watching for the legs not to be damaged) until it cracked off, and placed in a DNA extraction buffer. After extraction (see below), the abdomen was transferred to 10% KOH solution and genitalia were dissected in a standard manner. A leg was used for primary type specimens. A leg was removed from a specimen using fine forceps and placed in a plastic tube. The forceps were wiped with clean paper tissue after each sample was taken.

DNA was extracted from legs (and abdomens) non-destructively using Macherey-Nagel (MN) reagents. 70 µl buffer T1 and 10 µl protK were added to the tube without crushing the leg, and the mixture was incubated at 57 °C for 24 hours. Then, 80 µl buffer B3 was added and incubation continued for 2 hours, after which 85 µl of absolute EtOH was added and thoroughly mixed. The resulting liquid was transferred to a different tube and DNA extraction continued according to MN protocol (https://www.mn-net.com/Portals/8/attachments/Redakteure_Bio/Protocols/Genomic%20DNA/UM_gDNATissueXS.pdf), leaving the leg intact. Mate-pair libraries were constructed according to our published protocols (Cong et al. 2015; Cong et al. 2017; Li et al. 2019).

The libraries were sequenced for 150 bp from both ends targeting 4 to 6 Gbp of data (depending on the expected genome size) on Illumina HiSeq x10 at GENEWIZ. The resulting reads were matched using Diamond (Buchfink et al. 2015) to the exons of the reference genome of *Cecropteruslyciades* (Shen et al. 2017), which we obtained previously, and the exons assembled and aligned to other Hesperiidae genomes obtained using the same methods. Coding regions of the mitochondrial genome (including the COI barcode) were assembled similarly. Exons expected to be from the Z-chromosome were predicted assuming similar syntenic arrangement with *Heliconius* (Heliconius Genome Consortium 2012). Phylogenetic trees were generated from three sets of exons: whole nuclear genome, whole mitochondrial genome and Z-chromosome using RAxML-NG (Kozlov et al. 2018) with default parameters (-m GTRGAMMA). Further details of experimental and computational protocols can be found in the “SI Appendix” to Li et al. (2019) (available at https://www.pnas.org/content/pnas/suppl/2019/03/15/1821304116.DCSupplemental/pnas.1821304116.sapp.pdf).

Diagnostic DNA characters were identified in nuclear genomic sequences using our recently published procedure (see SI Appendix to Li et al. 2019). Namely, the positions in exons were found that are most likely synapomorphic to the clade defined as a subfamily. For the clades where we had several species sequenced, positions that were invariant in all species and had a base pair different from the (mostly invariant) base pair in the outgroups were found, and those with the smallest number of species with missing data were selected. If the subfamily had only one species sequenced, we frequently looked for synapomorphic characters for its sister, noting the base pair as the character state, and uniting these with synapomorphic characters for the clade that leads to the common ancestor of this subfamily and its sister clade. Such a treatment increased the chances that the character found is not a random, non-conserved change or a sequencing error. The number of sequence reads covering this position was taken into account in choosing the characters, and those positions with higher coverage were given priority. The character states are given in diagnoses below as abbreviations. For example, aly728.44.1:G672C means position 672 in exon 1 of gene 44 from scaffold 728 of *Cecropterus* [formerly *Achalarus*] *lyciades* (aly) reference genome (Shen et al. 2017) is C, changed from G in the ancestor. When characters were found for the sister clade of the diagnosed taxon, the following statement was used: aly5294.20.2:A548A (not C), which means that position 547 in exon 2 of gene 20 on scaffold 5294 is occupied by the ancestral base pair A, which was changed to C in the sister clade (so it is not C in the diagnosed taxon). 169A, means position 169 is A, but the ancestral state is unclear. The sequences of exons from the reference genome with the positions used as character states highlighted in green are given in the Suppl. material 1. The distribution of these sequences together with this publication ensures that the numbers given in the diagnoses can be easily associated with actual sequences. Notations like A79T or 59C, without scaffold.gene.exon prefix separated by colon, refer to positions in the standard COI barcode region of 658 positions as defined previously (Ratnasingham and Hebert 2007). The sequences reported in this paper have been deposited in the NCBI Sequence Read Archive with accession PRJNA544364.

## Results and discussion

### Genomic phylogeny of Hesperiidae

We obtained whole genome shotgun sequence reads for 160 Hesperiidae specimen of representative species. The lengths of resulting genomic regions were: nuclear total 11,835,126 +/-3,035,464, Z-chromosome 99,237 +/-24,462, mitogenomes 12,144 +/-958. We considered Z-chromosome separately. Butterfly males carry two copies of Z, and females possess Z and W. In Z, recombination is reduced to half of that in autosomes, and sexual selection acts differently on genes encoded by it. Thus, the analysis of genes encoded by the Z-chromosome may provide additional information about species evolution. Phylogenetic trees were constructed from coding regions of nuclear genome, Z-chromosome and mitogenome. The trees were rooted with the genomic sequence of *Pterourusglaucus* that we obtained previously (Cong et al. 2015). Comparison of these trees yielded the same conclusions.

Several conclusions confirmed previous findings (Warren et al. 2008; 2009; Sahoo et al. 2016; Sahoo et al. 2017; Zhang et al. 2017; Espeland et al. 2018; Toussaint et al. 2018; Li et al. 2019). (1) The subfamily Coeliadinae Evans, 1937 is sister to all other Hesperiidae; (2) Euschemonidae Kirby, 1897 branches off next; (3) Eudaminae is sister to Pyrginae; (4) Heteropterinae is sister to Trapezitinae with Hesperiinae; and (5) Groupings into tribes mostly agree with what is known about Hesperiidae. However, several findings were new and some were unexpected. Three cases were particularly interesting and were analyzed in detail, as follows.

### The *Katreus* and *Ortholexis* clade is a new subfamily

**Figure 2.**
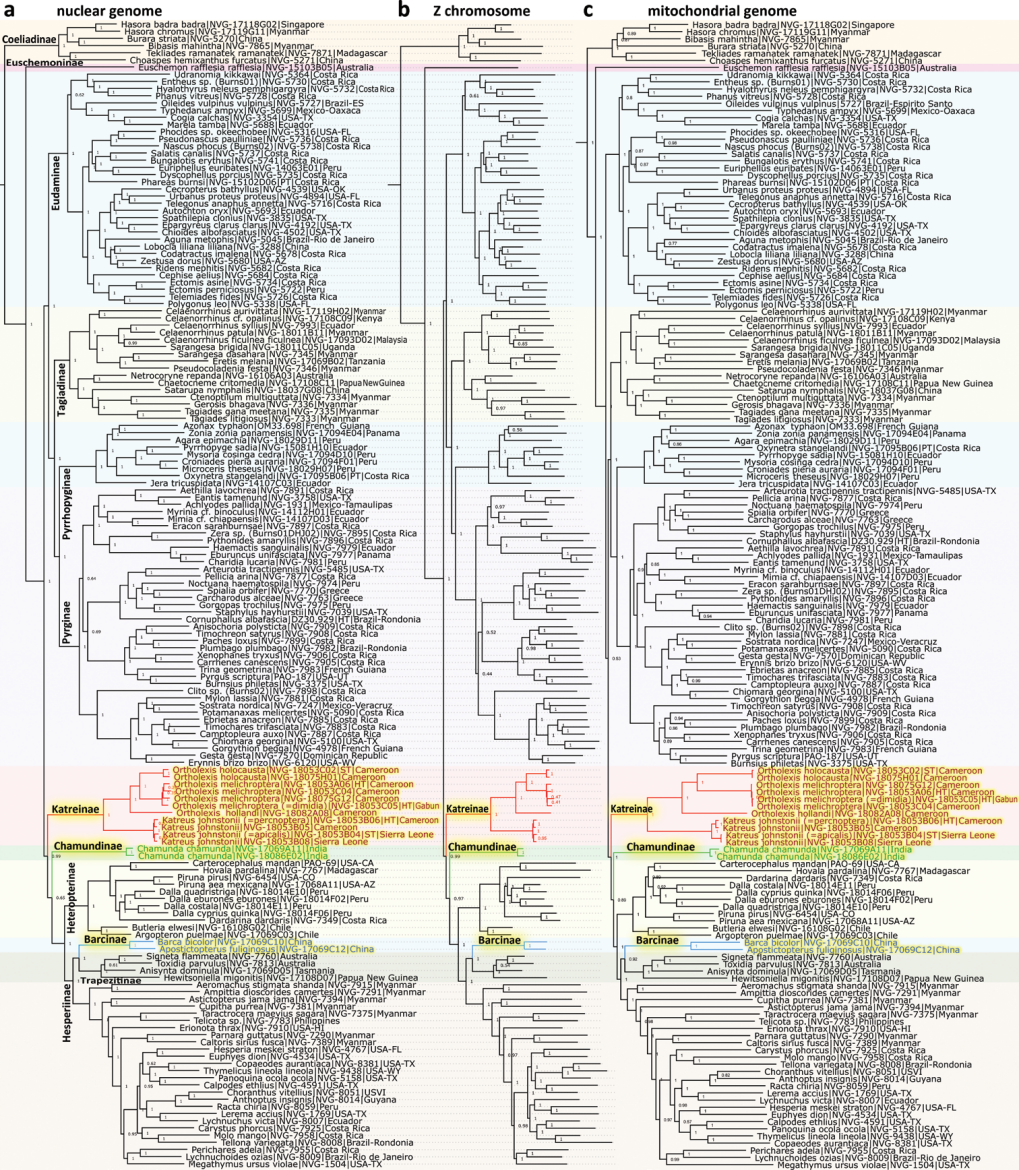
Phylogenetic trees. The trees are constructed from protein-coding regions of **a** nuclear genome **b** Z-chromosome, and **c** mitochondrial genome. The trees are rooted with *Pterourusglaucus* (NVG-1670). Specimen names are not shown in the Z-chromosome tree and can be deduced from the nuclear tree by corresponding dotted lines. Details about specimens are in Suppl. material 1: Table S1. Sections of the tree corresponding to different subfamilies are highlighted in different colors. Names of new subfamilies and specimens in them are highlighted yellow. Names of other subfamilies are shown by their clades in the nuclear tree.

Unexpected placement of *Ortholexisholocausta* (Mabille, 1891) (Fig. 1a) as a sister of Pyrrhopygini Mabille, 1877 in a recently published phylogeny of Hesperiidae based on several genes (Sahoo et al. 2017) peaked our interest about this taxon and its relatives. The genome-based phylogeny we obtained (Fig. 2) confidently (>99% bootstrap) places it (Fig. 1a–d), together with its sister genus *Katreus* Watson, 1893 (Fig. 1e), near the base of the Hesperiidae tree, dating prior to divergence between Eudaminae and Pyrginae (Fig. 2) and suggesting a rank of subfamily for these skippers.

#### Katreinae

Taxon classificationAnimaliaLepidopteraHesperiidae

Grishin subfam. n.

http://zoobank.org/EFD73E63-A0FE-4AB3-B6F2-318977EF7F83

##### Type genus.

*Katreus* Watson, 1893.

##### Diagnosis.

In appearance, most similar to *Celaenorrhinus* Hübner, [1819] and its relatives (Evans 1937), and was placed in Celaenorrhinini Swinhoe, 1912 by Warren et al. (2008, 2009) but differs by longer apiculus of antennae and hindwing produced at vein 1A+2A. Morphologically, distinguished from all Hesperiidae by the combination of the following characters. Abdomen short, shorter than inner margin of hindwing. Antennal club arcuate, bent in the middle, apiculus long, pointed. Second segment of palpi protrudes partly forward and partly upward (at an angle between the axis of the body and the axis perpendicular to it, =sub-erect). Males with hair pencil on hind tibiae, without stigmas or brands on wings. Forewing discal cell long, about 2/3 of the costa; vein M_2_ originates about midway between or closer to M_1_ than to M_3_ and vein CuA_2_ originates closer to the base of wing than to the end of discal cell. Hindwing produced at vein 1A+2A, vein 3A much shorter than vein CuA_2_. Male genitalia with a well-developed gnathos, which is not smaller than uncus, uncus bulging dorsad in lateral view, with small or tiny arms distant from each other, tegumen robust, extends caudad for the length of uncus, harpe longer than sacculus. See Larsen (2005: 469-471) for illustrations of all representative species in this subfamily. In DNA, a combination of the following base pairs in the nuclear genome is diagnostic: aly528.10.2:G940C, aly925.27.5:A3610T, aly84.77.5:T1651G, aly595.14.2:G184C, aly2284.22.2:G967C, and in COI barcode region: C235T, A335T, C347T, and T349A.

##### Genera included.

*Katreus* with its invalid synonym *Choristoneura* Mabille, 1889 (junior homonym of *Choristoneura* Lederer 1859 in Lepidoptera: Tortricidae) and subjective synonyms *Loxolexis* Karsch, 1895 and *Daratus* Lindsey, 1925 (replacement name for *Choristoneura*) (Fig. 1e); and *Ortholexis* Karsch, 1895 with its subjective synonym *Acallopistes* Holland, 1896 (Fig. 1a-d).

##### Comments.

Taxonomy of these skippers has been confusing until it was resolved by Cock and Congdon (2011). For the most part, they were all placed in the genus *Katreus*, until Larsen emphasized the differences in genitalia of those species placed in *Ortholexis* from true *Katreus* (Larsen 2005). Indeed, the two genera are quite distinct in our genomic analysis. A recent study based on several genes placed this group (only *Ortholexisholocausta* (Mabille, 1891) was included in that study) as a sister of Pyrrhopygini Mabille, 1877 (Sahoo et al. 2017), probably due to an insufficient number of genes included. In their study, *Euschemon* Doubleday, 1846 grouped with Eudaminae instead of being sister to all other Hesperiidae with exclusion of Coeliadinae Evans, 1937 (Warren et al. 2009; Zhang et al. 2017; Toussaint et al. 2018); such problems are expected from smaller datasets. We find (Fig. 2) that the Katreinae subfam. n. is an ancient and unique Afrotropical lineage that diverged from other Hesperiidae at the time when the family was diversifying into subfamilies.

### *Acallopistesdimidia* Holland, 1896 is a new subjective synonym of *Ortholexismelichroptera* Karsch, 1895

We sequenced a syntype of *Erionotaholocausta* Mabille, 1891 (Fig. 1a, judging from the original description (Mabille 1891) the type series of this species almost certainly consisted of this single syntype), and the holotypes of *Acallopistesdimidia* Holland, 1896 (Fig. 1c) and *Ortholexismelichroptera* Karsch, 1895 (Fig. 1d), which are in the Museum für Naturkunde, Berlin, Germany. The phylogenetic trees (Fig. 2) revealed that *O.melichroptera* is not a female of *O.holocausta* as it has been assumed (Evans 1937), but instead a female of *O.dimidia*. This association of sexes is supported by both nuclear (protein-coding genes of autosomes and of Z-chromosome) and mitochondrial (all genes) DNA trees (Fig. 2). COI barcodes of the *O.holocausta* syntype and *O.melichroptera* holotype differ by 8.8% (58 bp), but barcodes of *O.melichroptera* and *O.dimidia* are essentially identical (1 bp difference). Thus, we conclude that *O.dimidia* syn. n. is a junior subjective synonym of *O.melichroptera*.

### Unexpected uniqueness of *Chamunda*

The next find was particularly unexpected and was not likely to happen in the absence of DNA sequences. Nearly as ancient as Katreinae subfam. n., is the lineage consisting of a single genus *Chamunda* Evans, 1949, which is sister to the group collectively known as “grass skippers”: subfamilies Heteropterinae Aurivillius, 1925, Trapezitinae Waterhouse & Lyell, 1914 and Hesperiinae Latreille, 1809 (Fig. 2), whose caterpillars feed mostly on monocots. The surprise comes due to the fact that *Chamunda* looks like an ordinary skipper, quite similar to several others in wing patterns: brown with forewing white spots forming a typical arrangement for dicot-feeding skippers (Fig. 1f). Nevertheless, its ancient origin suggests a subfamily rank, as described below.

#### Chamundinae

Taxon classificationAnimaliaLepidopteraHesperiidae

Grishin subfam. n.

http://zoobank.org/4FE1725C-4BF1-4D1A-B4A7-4BD409AA154A

##### Type genus.

*Chamunda* Evans, 1949.

##### Diagnosis.

Keys to C.10 in Evans (1949: 14). In appearance similar to Pyrginae, such as *Celaenorrhinus* Hübner, [1819] and its relatives, from which it is distinguished by the second segment of palpi protruding forward (in line with the body, =porrect) and not pointing dorsad (perpendicular to the body line, =erect); and Eudaminae, such as *Lobocla* Moore, 1884, from which it differs by narrower hindwing without tornal lobe (concave outer margin near tornus) and the lack of costal fold in males. Morphologically, distinguished from all Hesperiidae by a combination of the following characters. Body robust, abdomen stout, shorter than the inner margin of hindwing. Palpi porrect, 3rd segment stout, pointing forward, set at the outer edge of the second segment (not in the middle). Antennae longer than half of costa, with thin arcuate (not hooked) club and apiculus tapered to a sharp point, nudum of about 20 segments. Males with hair pencil on hind tibiae, without stigmas or brands on wings. Females with anal tuft of scales. Forewing discal cell long, about 2/3 of the wing; vein M_2_ origin slightly closer to M_1_ than to M_3_. Five subapical spots in a S-shaped curve on right forewing. Hindwing inner margin shorter than costal margin; vein M_2_ straight and oblique: closer to M_3_ at the outer margin, but closer to M_1_ at its origin from the discal cell (not curved toward M_3_); the angle formed by the median and discocellular veins acute, discocellular vein directed at tornus and outer margin, and not at the inner margin. In male genitalia, uncus elongated, undivided, uniquely shaped like a narrow mushroom at the tip; valva simple, without processes, spines or elaborations, lanceolate, with a small harpe only narrowly separated from the ampulla. In DNA, a combination of the following base pairs in the nuclear genome is diagnostic: aly528.10.2:A631C, aly3277.11.2:A1726G, aly4523.3.2:T143C, aly499.37.1:G77G (not A), aly363.14.5:A76A (not C), aly2700.1.4:T70T (not G), and in COI barcode region: G38A, A81C, A307G, C347T, T349A, A430T, A604C.

##### Genera included.

Only *Chamunda*, a monotypic genus for *Plesioneurachamunda* Moore, 1866 (Fig. 1f).

##### Comments.

The subfamily-worthy uniqueness of this butterfly from southwestern Asia, dubbed “Olive” or “Crescent Spotted Flat”, is perhaps the largest surprise of our study. *Chamunda* is not clearly distinct in appearance, it is similar to *Lobocla* (Eudaminae) and *Celaenorrhinus* (Tagiadinae) in the spotting of the forewing. Uniqueness of *Chamunda* was not noticed before Evans, who established a new monotypic genus for this skipper (Evans 1949). Nevertheless, Evans placed it with Pyrginae according to its appearance, among genera currently in the tribe Tagiadini Mabille, 1878. We take the next step and establish a subfamily for it. It is unlikely that its subfamily status would have become apparent without genomic sequences placing this skipper far from all others with strong statistical support.

### The *Barca* and *Apostictopterus* clade originates near Trapezitinae and Hesperiina

These two genera that are apparently each other’s closest relatives have been enigmatic for decades (Evans 1949) (Fig. 1g, h). Their mitogenomes have recently been sequenced (Han et al. 2018) and revealed that among species with known mitogenomes (which did not include any Trapezitinae), they are sister to Hesperiinae and not Heteropterinae. Ironically, our study suggests that Trapezitinae may be sister to the group formed by these two genera (Fig. 2). However, no apparent morphological synapomorphies unify the group of the two genera with Trapezitinae, and their morphology is quite different, so we award them a subfamily rank:

#### Barcinae

Taxon classificationAnimaliaLepidopteraHesperiidae

Grishin subfam. n.

http://zoobank.org/A3512E6F-78AF-4AB5-9562-43C160BFA2D7

##### Type genus.

*Barca* de Nicéville, 1902.

##### Diagnosis.

Keys to F.4a in Evans (1949: 23). The synapomorphy of the subfamily is likely to be the bow-like shape of the forewing vein A_1_+A_2_. In appearance similar to Heteropterinae (slender body and characteristic relatively broad for monocot-feeding Hesperiidae but rounded wing shape), from which it is distinguished by this bowed vein and not flattened antennal club with obtuse apiculus. Morphologically, distinguished from all Hesperiidae, by the following combination of additional characters. Body slender, abdomen not longer than inner margin of hindwing. Second segment of palpi protruding forward (in line with the body, =porrect) and not pointing dorsad (perpendicular to the body line, =erect). Apiculus of antennae blunt, with black nudum of 10 segments, more than in Heteropterinae (6-9) but fewer than Trapezitinae (12-26). Mid tibiae without spines and hind tibiae with 2 pairs of short spurs. No secondary sexual characters. Forewing discal cell about 2/3 of costa in length, apex rounded. Hindwing with a rounded tornus, costal margin longer than inner margin; discal cell not shorter than half of the wing; discocellular vein points toward tornus, not inner margin. Male genitalia with extended, undivided uncus (Evans 1949: plate 29 F.4, F.5) more similar to Heteropterinae, but valva broader and more robust and reminiscent of that in Trapezitinae: expanded and modified costa-ampulla, harpe prominent, with serrated edge. In DNA, a combination of the following base pairs in the nuclear genome is diagnostic: aly525.83.3:A682T, aly525.83.3:G683C, aly1139.27.4:G112T, aly1139.27.4:G113C, aly23605.15.15:G49A, and in COI barcode region: G101A, A166G, and 474C.

##### Genera included.

*Barca* de Nicéville, 1902 with its invalid synonym *Dejeania* Oberthür, 1896 (junior homonym of *Dejeania* Robineau-Desvoidy, 1830 in Diptera) (Fig. 1g) and *Apostictopterus* Leech, [1893] with its subjective synonym *Tecupa* Swinhoe, 1917 (Fig. 1h). Both valid genera are monotypic.

##### Comments.

These two genera from southwestern China were (with disclaimers) placed in Heteropterinae by Evans (Evans 1949) and transferred to Hesperiinae by Warren et al. (2009), owing to different from Heteropterinae genitalia. Mitochondrial genomes for both genera were determined recently, and they confirmed the lack of affinity to Heteropterinae (Han et al. 2018). However, in the absence of Trapezitinae mitogenome, the two genera remained in Hesperiinae. Our phylogenies place the two genera as sister to Trapezitinae, thus they may not belong to Hesperiinae. This placement is unexpected because there are no obvious morphological features than unify Trapezitinae and the two genera. Therefore, we decided on the level of a subfamily for these two unusual skippers. They form an ancient phylogenetic group, and placing them within Trapezitinae seems unfitting due to the lack of morphological affinities.

### Phylogeny and classification

While classification relies on phylogeny, it does not require phylogeny to be fully resolved. Good classification only requires a clade itself to be well supported and distinct from other clades of the same rank. However, the exact position of that clade in the tree, which reflects the order in time when these clades originated, does not need to be fully resolved. Thus, accurate classification is a simpler task than phylogenetic inference. These considerations are relevant to our treatment of Chamundinae subfam. n. While in the Z-chromosome tree (Fig. 2b), the node at which Chamundinae have split from its sister is well supported (97% bootstrap), both nuclear genome and mitogenome trees (Fig. 2a, c) reveal weaker support: 65% and 89% respectively. It is likely that the weak support is a consequence of rapid radiation at the time of divergence between Katreinae subfam. n., Chamundinae and the sister of these taxa (the monocot feeding clade: Heteropterinae plus their sister clade). Possible incomplete lineage sorting and introgression obscured phylogenetic signal and leave the exact position of Chamundinae clade weakly resolved.

Nevertheless, the decision to treat Chamundinae as a subfamily is supported by the following reasons. We consider three clades in the trees (Fig. 2): Katreinae (colored red), Chamundinae (colored green), and the clade of monocot feeders (Heteropterinae plus their sister clade that includes Barcinae subfam. n., Trapezitinae and Hesperiinae). The clade of monocot feeders is well supported in all three trees (Fig. 2, bootstrap 100%), therefore Chamundinae does not belong to this clade. The clade of Katreinae plus their sister (Chamundinae, Heteropterinae, Barcinae, Trapezitinae, and Hesperiinae) is also strongly supported in all three trees (bootstrap >99%), therefore Chamundinae belong to this clade. The placement of the three subclades in this clade (Katreinae, Chamundinae and the monocot feeder) is poorly resolved. I.e., it is possible that: (1) Chamundinae are the sister to the clade consisting of the two others, or (2) Katreinae are the sister to the clade consisting of the two others (as in the trees in Fig. 2), or (3) Katreinae and Chamundinae are sister taxa. In all three scenarios, Chamundinae get the subfamily rank. In (1) & (2), Chamundinae originated prior to the split of their sister into subfamilies, so they should be a subfamily. If the scenario (3) is true, it would be conceivable to unify Katreinae and Chamundinae in a single subfamily, but the monophyly of this putative subfamily would be poorly supported (the same 65% bootstrap in nuclear genome tree). Therefore, because of this weak support, Chamundinae should receive the subfamily rank. Moreover, in the scenario (3), Katreinae and Chamundinae would have diverged from each other prior to divergence of the monocot-feeding clade into subfamilies, so each clade is more consistent with the subfamily rank.

## Conclusions

Genomics analysis has been instrumental in revealing the ancient origins of several groups of Hesperiidae that have not been understood before. Moreover, previous studies based on smaller DNA datasets, such as several genes (Sahoo et al. 2016; Sahoo et al. 2017) or mitochondrial genomes (Han et al. 2018) remained inconclusive. Whole genome shotgun reads assembled into protein-coding genes strongly support the uniqueness of the three groups of skippers dealt with in this study and indicate that these groups diverged from other Hesperiidae very early in the evolution of the family. Divergence times of Katreinae subfam. n. and Chamundinae subfam. n. from other Hesperiidae are earlier than the split of the ancestors of subfamilies Heteropterinae and Trapezitinae. Deep divergence times argue for the subfamily status of these groups. Subfamily Barcinae subfam. n. unexpectedly emerges as a possible sister of Trapezitinae, but is morphologically quite different from them. Whole genome shotgun sequencing was instrumental for this study. Notably, our methods are equally applicable to specimens kept in collections for more than a century. Sequencing of the primary type specimens collected over 120 years ago establishes sex association for the species with extreme sexual dimorphism. As a result, a new synonymy is introduced, and the species known before as *Ortholexisdimidia* should be referred to as *Ortholexismelichroptera*.

## Supplementary Material

XML Treatment for Katreinae

XML Treatment for Chamundinae

XML Treatment for Barcinae

## Supplementary materials

Supplementary material 1 Table S1. Specimen data and DNA sequences Data type: table, text and DNA sequences Explanation note: Table S1 with data for 160 sequenced specimens and diagnostic nucleotide characters of the new subfamilies mapped to the reference genome of *Cecropteruslyciades*. This dataset is made available under the Open Database License (http://opendatacommons.org/licenses/odbl/1.0/). The Open Database License (ODbL) is a license agreement intended to allow users to freely share, modify, and use this Dataset while maintaining this same freedom for others, provided that the original source and author(s) are credited.Jing Zhang, Qian Cong, Jinhui Shen, Ernst Brockmann, Nick V. Grishin
