# Supplementary material for: Three new subfamilies of skipper butterflies (Lepidoptera, Hesperiidae)
Source: Zookeys. 2019 Jul 8;861:91–105. doi: 10.3897/zookeys.861.34686 (PMC6629708; doi:10.3897/zookeys.861.34686)
Supplement: Supplementary material 1 [file zookeys-861-091-s001.pdf]

Table S1. Data for 160 sequenced Hesperiidae specimens

#	DNA Voucher	Taxon name	Type	Sex	Locality	Elevation	GPS	Collectors	Date	Collection	Genitalia No.	Collection No.	Molecular	No. of positions*
1	NVG-17118G02	***Hasora badra badra***		F	Singapore: MacRitchie Reservoir			Stephen Kinyon	22-Apr-1989	USNM				9833229, 66918, 12744
2	NVG-17119G11	***Hasora chromus***		M	Myanmar: S. Shan State, Pay Ne Bin, near Kalaw			Stephen Kinyon	30-May-2002	USNM				12764836, 107430, 12573
3	NVG-5270	***Burara striata***		F	China: Sichuan Prov., Pingwu Co.			Rongjiang Wang	11-Aug-2015					12513596, 100104, 8488
4	NVG-7865	***Bibasis mahintha***		F	Myanmar: S. Shan State, Kalaw	1500 m	20.61500, 96.57667	Stephen Kinyon	29-Sep-2001	USNM	NVG170206-50			7517016, 47403, 12376
5	NVG-7871	***Tekliades ramanatek ramanatek***		M	Madagascar: Prov. Fianarantsoa, 7 km W Ranomafana	900 m		W. E. Steiner	20-31-Jan-1990	USNM	NVG170206-56			9910363, 89493, 12727
6	NVG-5271	***Choaspes hemixanthus furcata***			China: Sichuan Prov., Pingwu Co.			Rongjiang Wang	12-Aug-2015					10603967, 78433, 7145
7	NVG-15103B05	***Euschemon rafflesia rafflesia***		M	Australia: Queensland, Southbrook			maybe C. B. Davidson	probably around 1946	USNM				4593703, 43461, 12629
8	NVG-5364	***Udranomia kikkawai***		F	Costa Rica: Guanacaste Prov., ACG	710 m	10.98445, -85.42481	Calixto Moraga	eclosed on 28-Apr-2014	USNM		14-SRNP-30490		13357869, 106983, 8329
9	NVG-5730	***Entheus* sp.** (Burns01)		F	Costa Rica: Guanacaste Prov., ACG	1220 m	10.92918, -85.46426	Winnie Hallwachs	eclosed on 28-Jul-2008	USNM	NVG160214-91	08-SRNP-35619		12982120, 88737, 12282
10	NVG-5732	***Hyalothyrus neleus pemphigargyra***			Costa Rica: Guanacaste Prov., ACG	290 m	11.03226, -85.52776	Lucia Ríos	eclosed on 6-Sep-2013	USNM	NVG160214-93	13-SRNP-21784		14456386, 120015, 13256
11	NVG-5728	***Phanus vitreus***			Costa Rica: Alajuela Prov., ACG	645 m	10.87766, -85.39343	Elda Araya	eclosed on 22-Oct-2008	USNM	NVG160214-89	08-SRNP-5260		13171284, 112859, 12549
12	NVG-5727	***Oileides vulpinus***		M	Brazil: Espírito Santo, 25 km, N Linhares			Stan S. Nicolay	6-May-1969	USNM	NVG160214-88			9486980, 84912, 12700
13	NVG-5699	***Typhedanus ampyx***		M	Mexico: Oaxaca, Zipolite	sea level		John Kemner	11-Sep-1992	USNM	NVG160214-60			10916373, 94419, 11402
14	NVG-3354	***Cogia calchas***		M	USA: TX, Hidalgo Co.		26.07093, -97.89131	Qian Cong & Nick V. Grishin	23-May-2015					14455104, 119844, 12412
15	NVG-5688	***Marela tamba***		M	Ecuador: Pastaza, 8 km S Shell		-1.56666, -78.03333	Stan S. Nicolay	20-Oct-1991	USNM	NVG160214-49			12055037, 99283, 11512
16	NVG-5316	***Phocides* sp. *okeechobee***		M	USA: FL, Monroe Co.			Nick V. Grishin	19-Dec-2015					15199040, 123606, 12326
17	NVG-5736	***Pseudonascus paulliniae***			Costa Rica: Alajuela Prov., ACG	96 m	10.96187, -85.28045	Keiner Aragón	eclosed on 31-Oct-2013	USNM	NVG160214-97	13-SRNP-79622		13205866, 87213, 13229
18	NVG-5738	***Nascus phocus*** (Burns02)		M	Costa Rica: Guanacaste Prov., ACG	420 m	10.74160, -85.42734	Jose Cortez	eclosed on 15-Oct-2007	USNM	NVG160214-99	07-SRNP-60000		6335858, 52173, 10633
19	NVG-5737	***Salatis canalis***			Costa Rica: Guanacaste Prov., ACG	440 m	10.98670, -85.38503	Ricardo Calero	eclosed on 15-Apr-2013	USNM	NVG160214-98	13-SRNP-70310		14588744, 119166, 12248
20	NVG-5741	***Bungalotis erythus***		M	Costa Rica: Alajuela Prov., ACG	320 m	11.01227, -85.34929	Duvalier Briceño	eclosed on 2-May-2008	USNM	NVG160217-02	08-SRNP-65224		14970685, 121104, 12022
21	NVG-14063E01	***Euriphellus euribates***		M	Peru: Madre de Dios, Amazonia Lodge	491 m		Stephen Kinyon	24-Oct-2013	USNM				12871080, 105696, 10092
22	NVG-5735	***Dyscophellus porcius***		M	Costa Rica: Alajuela Prov., ACG	123 m	10.93548, -85.25314	Keiner Aragon	eclosed on 30-Aug-2013	USNM	NVG160214-96	13-SRNP-76944		14447632, 118863, 12880
23	NVG-15102D06	***Phareas burnsi***	PT		Costa Rica: Guanacaste Prov., ACG	440 m	11.01926, -85.40997	Petrona Rios	eclosed on 20-Mar-2005	USNM	NVG120513-05	05-SRNP-30577		15201344, 122811, 10600
24	NVG-4539	***Cecropterus bathyllus***		F	USA: OK, Atoka Co.		34.41040, -95.91059	Nick V. Grishin	22-Aug-2015					15328369, 116856, 12340
25	NVG-4894	***Urbanus proteus proteus***			USA: FL, Miami-Dade Co.		25.44190, -80.43996	Nick V. Grishin	4-Oct-2015					15360905, 122403, 12658
26	NVG-5716	***Telegonus anaphus annetta***			Costa Rica: Alajuela Prov., ACG	660 m	10.77824, -85.39458	Keiner Aragon	eclosed on 3-Feb-2010	USNM	NVG160214-77	10-SRNP-67040		15300654, 122673, 12665
27	NVG-5693	***Autochton oryx***		M	Ecuador: Sucumbios, Cerro Lumbaqui Norte	800-950 m	0.02222, -77.32277	Jason P. W. Hall & M. Alma Solis	18-22-Aug-2002	USNM	NVG160214-54			15064749, 121632, 12922
28	NVG-3835	***Spathilepia clonius***		M	USA: TX, Starr Co.		26.40521, -99.01941	Qian Cong & Nick V. Grishin	28-Jun-2015					15415053, 123753, 11255
29	NVG-4192	***Epargyreus clarus clarus***		F	USA: TX, Dallas Co.		32.84512, -96.71805	Nick V. Grishin	21-Jul-2015					15226596, 121413, 12939
30	NVG-4502	***Chioides albofasciatus***		M	USA: TX, Cameron Co.			Nick V. Grishin	16-Aug-2015					14133931, 121929, 10358
31	NVG-5045	***Aguna metophis***		F	Brazil: Rio de Janeiro, Guapimirim	50 m	-22.53333, -42.98333	Robert K. Robbins & Marcelo Duarte	17-Dec-1996	USNM	NVG151101-96			14881083, 109810, 10571
32	NVG-3288	***Lobocla liliana liliana***			China: Yunnan, Mengla Co.				Jun-2009					13271835, 116388, 9723
33	NVG-5678	***Codatractus imalena***			Costa Rica: Alajuela Prov., ACG	722 m	10.86546, -85.39694	Elda Araya	eclosed on 28-May-2012	USNM	NVG160214-39	12-SRNP-1823		15170703, 115773, 11826
34	NVG-5680	***Zestusa dorus***		M	USA: AZ, Coconino Co.	1500 m		Ron H. Leuschner	3-May-1984	USNM	NVG160214-41			15140351, 122673, 12696
35	NVG-5682	***Ridens mephitis***			Costa Rica: Guanacaste Prov., ACG	290 m	11.03226, -85.52776	Lucia Ríos	eclosed on 19-Apr-2014	USNM	NVG160214-43	14-SRNP-20297		14860977, 120807, 12539
36	NVG-5684	***Cephise aelius***			Costa Rica: Guanacaste Prov., ACG	440 m	10.9867, -85.38503	Ricardo Calero	eclosed on 18-Jun-2014	USNM	NVG160214-45	14-SRNP-70854		15143553, 114981, 12745
37	NVG-5734	***Ectomis asine***			Costa Rica: Alajuela Prov., ACG	160 m	10.95991, -85.28298	Keiner Aragón	eclosed on 23-Nov-2013	USNM	NVG160214-95	13-SRNP-79769		15117798, 122706, 12450
38	NVG-5722	***Ectomis perniciosus***		M	Peru: Atalaya, Amazonia Lodge	491 m		Stephen Kinyon	25-Oct-2013	USNM	NVG160214-83			9244714, 80283, 12959
39	NVG-5726	***Telemiades fides***			Costa Rica: Guanacaste Prov., ACG	255 m	11.03028, -85.54781	Elieth Cantillano	eclosed on 14-Jun-2011	USNM	NVG160214-87	11-SRNP-20768		15181292, 123276, 12047
40	NVG-5338	***Polygonus leo***		M	USA: FL, Monroe Co.			Nick V. Grishin	19-Dec-2015					15098019, 122679, 12346
41	NVG-17119H02	***Celaenorrhinus aurivittata***		M	Myanmar: S. Shan State, Paya Gyi Gon			Stephen Kinyon	4-Jun-2002	USNM				12286683, 107019, 12839
42	NVG-17108C09	***Celaenorrhinus* cf. *opalinus***		F	Kenya: Kakamega Dist., Kakamega Forest		0.24000, 34.86000	R. R. Snelling	1-6-May-2001	LACM				9550598, 64221, 12379
43	NVG-7993	***Celaenorrhinus syllius***			Ecuador: Sucumbios, Cerro Lumbaqui Norte	950 m	0.02833, -77.32033	Jason P. W. Hall & M. Alma Solis	1-3-Jan-2002	USNM	NVG170207-78	USNMENT 01321833		7167574, 65658, 11113
44	NVG-18011B11	***Celaenorrhinus patula***		M	Myanmar: Rakhine Div., GwaTwsp Ye Phya			Auna Gyi	2-9-May-2003	USNM				8763765, 80406, 12720
45	NVG-17093D02	***Celaenorrhinus ficulnea ficulnea***			Malaysia: Borneo, Mount Kinabalu	1500-1800 m		A. D. Dodge & G. A. Goss	no date, old	USNM				4210621, 42414, 12488
46	NVG-18011C05	***Sarangesa brigida***		F	Uganda: Entebbe			R. Carcasson	Jun-1957	USNM				4615540, 28605, 12750
47	NVG-7345	***Sarangesa dasahara***		M	Myanmar: Mandalay Div., Alaungdaw Khattapa Nat. Park	300 m	22.32167, 94.48667	Stephen Kinyon	21-Sep-2001	USNM	NVG161105-18			8907933, 79497, 10971
48	NVG-17069B02	***Eretis melania***			Tanzania: Tanganyika, Usambara			G. Pringle	Aug-1963	USNM		USNMENT 01321969		4374077, 44601, 12693
49	NVG-7346	***Pseudocoladenia festa***		M	Myanmar: S. Shan State, Ywangan Twsp			Aung Hoe	17-25-May-2003	USNM	NVG161105-19			12536597, 88431, 12264
50	NVG-16106A03	***Netrocoryne repanda***		M	Australia: Queensland, Killarney			E. B. Skreen	28-Jan-1963	LACM				10491329, 97185, 12687
51	NVG-17108C11	***Chaetocneme critomedia critomedia***		M	Papua New Guinea: Ambunti			Trevor Lundstrom	9-May-1972	LACM				13855462, 88401, 12702
52	NVG-18037G08	***Satarupa nymphalis***			China : Sichuan, Luzhou				Jul-2010					13603656, 115353, 12498
53	NVG-7334	***Ctenoptilum multiguttata***		M	Myanmar: Chin State, Alaungdaw Khattapa Nat. Park			Stephen Kinyon	31-Jan-15-Feb-2001	USNM	NVG161105-07			11085978, 95061, 12437
54	NVG-7336	***Gerosis bhagava***		M	Myanmar: Rakhine Div., GwaTwsp Kan Tha Yar			Stephen Kinyon	2-9-May-2003	USNM	NVG161105-09			11501060, 96786, 10710
55	NVG-7335	***Tagiades gana meetana***		M	Myanmar: Rakhine Division, GwaTwsp Ye Phya			Aung Gyi	2-9-May-2003	USNM	NVG161105-08	USNMENT 01321280		12511538, 110353, 11322
56	NVG-7333	***Tagiades litigiosus***		M	Myanmar: S. Shan State, Kalaw Reservoir	1300 m	20.60335, 96.52999	Stephen Kinyon	30-Sep-2001	USNM	NVG161105-06			13407918, 116121, 10042
57	OM33.698	***Azonax typhaon***		M	French Guiana: Montagne de Kaw			Gallard	9-Apr-1993	OM-DZUP				14628349, 114261, 12725
58	NVG-17094E04	***Zonia zonia panamensis***		M	Panama: Canal Zone, Madden Forest Preserve			G. B. Small	23-Jul-1969	USNM		USNMENT 00894779		12405474, 97251, 12435
59	NVG-18029D11	***Agara epimachia***		M	Peru: Madre de Dios, Atalaya	491 m		Stephen Kinyon	27-Sep-2014	USNM				12749214, 115107, 12432
60	NVG-15081H10	***Pyrrhopyge sadia***		M	Ecuador: Zamora-Chinchipe, Chicana	1000 m		J.-C. Petit	29-Sep-2016	Brockmann				11655597, 105921, 12722
61	NVG-17094D10	***Mysoria cosinga cedra***		M	Peru: Cuzco, Paucartambo/Acianaco Rd.	3150 m		Stephen Kinyon	3-Nov-2016	USNM		USNMENT 00894773		13670634, 118131, 10946
62	NVG-17094F01	***Croniades pieria auraria***			Peru: Cuzco, Cosnipata Valley	1194 m		Stephen Kinyon	24-Oct-2016	USNM		USNMENT 00894758		14469538, 121296, 11348
63	NVG-18029H07	***Microceris theseus***		M	Peru: Cuzco, Cosnipata Valley	1050 m		Stephen Kinyon	1-Nov-2016	USNM				12682607, 115584, 10379
64	NVG-17095B06	***Oxynetra stangelandi***	PT	M	Costa Rica: Guanacaste Prov., ACG	1220 m	10.92918, -85.46426	Harry Ramirez	eclosed on 25-Aug-2002	USNM		02-SRNP-23283		14485679, 114999, 12437
65	NVG-14107C03	***Jera tricuspidata***		M	Ecuador: Loja, 6 km Catamayo-Loja Rd	1780 m	-3.99933, -79.31850	David H. Ahrenholz	16-Jan-2002	USNM	X-5455, J.M.Bruns 2003			14329868, 109424, 11064
66	NVG-7891	***Aethilla lavochrea***			Costa Rica: Guanacaste Prov., ACG	290 m	11.03226, -85.52776	Lucia Ríos	eclosed on 14-Oct-2013	USNM	NVG170206-76	13-SRNP-22231		12714638, 110229, 12864
67	NVG-3758	***Eantis tamenund***		F	USA: TX, Hidalgo Co.		26.06665, -97.88366	Qian Cong & Nick V. Grishin	27-Jun-2015					14448945, 112383, 12438
68	NVG-1931	***Achlyodes pallida***		M	Mexico: Tamaulipas, Villa Gomez Farias	500 m		Roy O. Kendall & C. A. Kendall	27-Jan-1974	TAMU	NVG140104-70			13793583, 116862, 12018
69	NVG-14112H01	***Myrinia* cf. *binoculus***		M	Ecuador: Napo, Yasuni National Park			Charles Bordelon & Edward Knudson	20-Sep-4-Oct-2003	TLS				12430454, 105530, 12670
70	NVG-14107D03	***Mimia* cf. *chiapaensis***		M	Ecuador: Pichincha Prov., Maquipucuna	1600 m		R. A. Raguso	25-Aug-1989	USNM	X-6055 J. M. Burns 2004			14120115, 118620, 12478
71	NVG-7897	***Eracon sarahburnsae***			Costa Rica: Alajuela Prov., ACG	96 m	10.96187, -85.28045	Minor Carmona	eclosed on 25-Oct-2010	USNM	NVG170206-82	10-SRNP-67957		12762703, 109902, 12379
72	NVG-7895	***Zera* sp.** (Burns01DHJ02)			Costa Rica: Guanacaste Prov., ACG			Dunia Garcia	eclosed on 22-Aug-2013	USNM	NVG170206-80	13-SRNP-35491		10837796, 98103, 12536
73	NVG-7896	***Pythonides amaryllis***			Costa Rica: Alajuela Prov., ACG	460 m	10.9163, -85.37869	Anabelle Cordoba	eclosed on 11-Oct-2006	USNM	NVG170206-81	06-SRNP-7674		12121389, 107643, 12816
74	NVG-7979	***Haemactis sanguinalis***		M	Ecuador: Napo Prov., Jatun Sacha Biological Station	450 m	-1.06666, -77.60000	David H. Ahrenholz	30-Sep-1991	USNM	NVG170207-64	USNMENT 01321819		9486387, 87987, 11917
75	NVG-7977	***Eburuncus unifasciata***			Panama: Darien Prov., Cana	500 m		Gordon B. Small	2-Jan-1984	USNM	NVG170207-62	USNMENT 01321817		11642935, 106425, 12928
76	NVG-7981	***Charidia lucaria***		F	Peru: Cuzco, Cosnipata Road	1450 m		Stephen Kinyon	12-Oct-2008	USNM	NVG170207-66	USNMENT 01321821		13146832, 101424, 12506
77	NVG-5485	***Arteurotia tractipennis tractipennis***		M	USA: TX, Hidalgo Co.			Nadine M. McGuire	2-Sep-1972	TAMU	NVG160110-26			9270191, 81126, 12162
78	NVG-7877	***Pellicia arina***			Costa Rica: Alajuela Prov., ACG	96 m	10.96187, -85.28045	Keiner Aragón	eclosed on 18-Mar-2013	USNM	NVG170206-62	13-SRNP-67590		13357915, 113898, 12263
79	NVG-7974	***Noctuana haematospila***		M	Peru: Cuzco, Cosnipata Road	1700 m		Stephen Kinyon	2-Feb-2011	USNM	NVG170207-59	USNMENT 01321814		14298798, 119337, 12379
80	NVG-7770	***Spialia orbifer***		F	Greece: Epirus				16-Jun-1992	USNM	NVG170205-55	USNMENT 01321610		11374845, 82053, 12689
81	NVG-7763	***Carcharodus alceae***		M	Greece: Olympia			Ron Leuschner	20-Oct-1990	USNM	NVG170205-48	USNMENT 01321603		8890136, 86079, 12788
82	NVG-7975	***Gorgopas trochilus***			Peru: Cuzco, Cosnipata Road	564 m		Stephen Kinyon	15-Oct-2008	USNM	NVG170207-60	USNMENT 01321815		12233829, 107739, 12295
83	NVG-7039	***Staphylus hayhurstii***		F	USA: TX, Delta Co.		33.28242, -95.82520	Qian Cong, Jing Zhang & Nick V. Grishin	24-Sep-2016					13625137, 101439, 12493
84	DZ30.929	***Cornuphallus albafascia***	HT	M	Brazil: Rondonia, linea 10 at Rio Pardo, off B-65 5 km S of Cacaulandia			G. T. Austin	18-Aug-1993	DZUP	GTA-3672			10084417, 87105, 12150

85	NVG-7909	***Anisochoria polysticta***		M	Costa Rica: Guanacaste Prov., ACG	280 m	10.85827, -85.61089	Ruth Franco	eclosed on 09-Dec-2004	USNM	NVG170206-94	04-SRNP-15751		13364406, 115623, 11437
86	NVG-7908	***Timochreon satyrus***			Costa Rica: Guanacaste Prov., ACG	420 m	10.74160, -85.42734	Jose Alberto Sanchez	eclosed on 13-Sep-2007	USNM	NVG170206-93	07-SRNP-58884		12063003, 106422, 12867
87	NVG-7899	***Paches loxus***		M	Costa Rica: Guanacaste Prov., ACG	280 m	11.02865, -85.48669	Freddy Quesada	eclosed on 05-Dec-2003	USNM	NVG170206-84	03-SRNP-30995		12625583, 109617, 12139
88	NVG-7982	***Plumbago plumbago***			Brazil: Rondonia, 8 km N Cacaulandia		-10.50000, -62.86666	David H. Ahrenholz	6-Nov-1989	USNM	NVG170207-67	USNMENT 01321822		9980269, 94503, 12419
89	NVG-7906	***Xenophanes tryxus***		M	Costa Rica: Alajuela Prov., ACG	575 m	10.88009, -85.38887	J. D. Turner & N. Turner	26-Mar-2010	USNM	NVG170206-91	10-SRNP-103428		12246057, 108691, 12773
90	NVG-7905	***Carrhenes canescens***			Costa Rica: Alajuela Prov., ACG	645 m	10.87766, -85.39343	Carolina Cano	eclosed on 10-Apr-2014	USNM	NVG170206-90	14-SRNP-1649		12556748, 87525, 12602
91	NVG-7983	***Trina geometrina***			French Guiana: Saul	200-450 m	3.61666, -53.71666	Don J. Harvey	19-Nov-1993	USNM	NVG170207-68	USNMENT 01321823		9632778, 86619, 12625
92	PAO-187	***Pyrgus scriptura***			USA: UT, Garfield Co.			Paul A. Opler and Evi Buckner-Opler	5-Aug-2016					13553006, 117024, 11780
93	NVG-3375	***Burnsius philetas***		M	USA: TX, Starr Co.		26.42298, -99.03123	Qian Cong & Nick V. Grishin	23-May-2015					14277133, 121101, 12677
94	NVG-7898	***Clito* sp.** (Burns02)			Costa Rica: Guanacaste Prov., ACG	280 m	11.03534, -85.53645	Roster Moraga	eclosed on 22-Aug-2013	USNM	NVG170206-83	13-SRNP-21146		14310277, 116889, 12649
95	NVG-7881	***Mylon lassia***			Costa Rica: Guanacaste Prov., ACG	1150 m	10.92691, -85.46822	Manuel Pereira	eclosed on 29-Aug-2009	USNM	NVG170206-66	09-SRNP-36601		11739028, 101931, 12388
96	NVG-7247	***Sostrata nordica***		M	Mexico: Veracruz, Santa Rosa			Collection Wm. Schaus	Aug-1906	USNM	NVG161005-74			12298888, 106536, 12869
97	NVG-5090	***Potamanaxas melicertes***			Costa Rica: Guanacaste Prov., ACG	675 m	10.98931, -85.42581	Freddy Quesada	eclosed on 08-Nov-2013	USNM	NVG151101-98	13-SRNP-31335		14025378, 118746, 12419
98	NVG-7885	***Ebrietas anacreon***			Costa Rica: Guanacaste Prov., ACG	290 m	11.02364, -85.49139	Elieth Cantillano	eclosed on 22-Nov-2006	USNM	NVG170206-70	06-SRNP-23020		12327003, 105495, 12655
99	NVG-7883	***Timochares trifasciata***			Costa Rica: Guanacaste Prov., ACG	70 m	10.86336, -85.72535	Guillermo Pereira	eclosed on 24-Mar-2005	USNM	NVG170206-68	05-SRNP-12097		8327443, 75576, 13030
100	NVG-7887	***Camptopleura auxo***			Costa Rica: Alajuela Prov., ACG	410 m	10.90425, -85.28651	Jose Perez	eclosed on 27-Aug-2012	USNM	NVG170206-72	12-SRNP-43606		11161967, 73809, 12724
101	NVG-5100	***Chiomara georgina***		F	USA: TX, Starr Co.		26.37038, -98.80675	Nick V. Grishin	14-Nov-2015					13209288, 98260, 12275
102	NVG-4978	***Gorgythion begga***		M	French Guiana: Montravel		4.91667, -52.26666	Don J. Harvey	4-Dec-1993	USNM	NVG151101-29			12141100, 105612, 12293
103	NVG-7570	***Gesta gesta***		M	Dominican Republic: 14 km NW of Jarabacoa			Everard M. Kinch	23-Jun-1981	TAMU	NVG170107-26			12878777, 112020, 12781
104	NVG-6120	***Erynnis brizo brizo***		F	USA: WV, Pendleton Co.		38.73398, -79.48996	Qian Cong & Nick V. Grishin	8-May-2016					14097645, 111441, 12596
105	NVG-18053C02	***Ortholexis holocausta***	ST	M	Cameroon: Barombi Station			Paul Preuss	1889	ZMHB				7230780, 73155, 12546
106	NVG-18075H01	***Ortholexis holocausta***		M	Cameroon: Bascho			Arnold Schultze	7-Mar-1906	ZMHB				7626492, 74877, 12587
107	NVG-18053A06	***Ortholexis melichroptera***	HT	F	Cameroon: Victoria			Preuss S.	prior to 1895	ZMHB				6581705, 45147, 12513
108	NVG-18053C04	***Ortholexis melichroptera***		M	Cameroon: Victoria			Paul Preuss	no date, old	ZMHB				2719729, 27312, 12723
109	NVG-18075G12	***Ortholexis melichroptera***		F	Cameroon: Bascho			Arnold Schultze	5-Mar-1906	ZMHB				5099430, 34038, 12612
110	NVG-18053C05	***Ortholexis melichroptera*** (=***dimidia***)	HT	M	Gabon				1890	ZMHB				6847332, 66396, 11316
111	NVG-18082A08	***Ortholexis hollandi***		M	Cameroon: Bitje				no date, old	BMNH		NHMUK_012824103	0247278421	5083263, 47916, 12686
112	NVG-18053B06	***Katreus johnstonii*** (=***percnoptera***)	HT	M	Cameroon: Barombi Station			Paul Preuss	prior to 1895	ZMHB				8516976, 61812, 12520
113	NVG-18053B05	***Katreus johnstonii***		F	Cameroon: Barombi Station			Paul Preuss	1889	ZMHB				9337696, 66897, 12696
114	NVG-18053B04	***Katreus johnstonii*** (=***apicalis***)	ST	M	Sierra Leone			Paul Preuss	prior to 1889	ZMHB				4876942, 51879, 12674
115	NVG-18053B08	***Katreus johnstonii***		F	Sierra Leone			Paul Preuss	1887	ZMHB				8544563, 57582, 12623
116	NVG-17069A11	***Chamunda chamunda***		M	India			G. C. Dudgeon	no date, old	USNM				3569218, 36507, 12396
117	NVG-18086E02	***Chamunda chamunda***		F	India: Sikkim				1905	MNHP		EL63205		5167561, 35385, 12356
118	PAO-69	***Carterocephalus mandan***			USA: CA, Sierra Co.			Paul A. Opler and Evi Buckner-Opler	19-Jun-2016					14035685, 117669, 12766
119	NVG-7767	***Hovala pardalina***		F	Madagascar: Fianarantsoa Prov., 7 km SW Ranomafana	1150 m		C. Kremen, R. Van Epps	13-Sep-1988	USNM	NVG170205-52	USNMENT 01321607		12036862, 99858, 12499
120	NVG-7349	***Dardarina dardaris***			Costa Rica: Guanacaste Prov., ACG	17 m	10.84918, -85.77315	Freddy Quesada	eclosed on 2-Aug-2004	USNM	NVG161105-22	04-SRNP-13073		11323426, 103134, 12424
121	NVG-6454	***Piruna pirus***		M	USA: CO, Grand Co.	2563 m	[illegible]0.02139, -106.07003	Nick V. Grishin	6-Jul-2016					13522168, 115611, 12883
122	NVG-17068A11	***Piruna aea mexicana***			USA: AZ, Santa Cruz Co.			Ken Davenport	27-Aug-2016	CSUC		CSU_ENT1033276		13219803, 114348, 12626
123	NVG-18014E10	***Dalla quadristriga***		M	Peru: Cuzco, Cosnipata Rd.	3320 m		Stephen Kinyon	26-Jan-2013	USNM				14418803, 120024, 12692
124	NVG-18014F02	***Dalla eburones eburones***		M	Peru: Cuzco, Cosnipata Rd.	2200 m		Stephen Kinyon	8-Nov-2008	USNM				13336994, 117439, 12697
125	NVG-18014E11	***Dalla costala***		M	Peru: Cuzco, Cosnipata Rd.	2200 m		Stephen Kinyon	13-Feb-2011	USNM				13986172, 118581, 12723
126	NVG-18014F06	***Dalla cyprius quinka***		M	Peru: Cuzco, Cosnipata Rd.	1450 m		Stephen Kinyon	2-Feb-2013	USNM				13114574, 112788, 12887
127	NVG-16108G02	***Butleria elwesi***		M	Chile: Llanquihue Province, Hornohuinco	300 m		D. R. Davis	29-21-Dec-1981	USNM				14745442, 103619, 12771
128	NVG-17069C03	***Argopteron puelmae***		M	Chile: Nuble, Las Trancas	1100 m		C. M. & O. S. Flint, Jr.	6-7-Feb-1987	USNM		USNMENT 01321981		13482588, 117135, 12731
129	NVG-17069C10	***Barca bicolor***		M	China: Sichuan province, Ta-Tsien-Lou				1910	USNM				3789671, 66126, 12697
130	NVG-17069C12	***Apostictopterus fuliginosus***		M	China: Sichuan province, Mount Emei			D. C. Graham	no date, old	USNM				9546128, 116886, 12714
131	NVG-7760	***Signeta flammeata***		M	Australia: New South Wales, Brown Mountain			Fred Sattler	16-Feb-1985	USNM	NVG170205-45	USNMENT 01321600		12470688, 121476, 12640
132	NVG-7813	***Toxidia parvulus***			Australia: Queensland, Crowns Nest National Park			C. Goospasture	29-Feb-1984	USNM	NVG170205-98			12449153, 122544, 11518
133	NVG-17069D05	***Anisynta dominula***		F	Australia: Tasmania, Cranbrook			S. Angel	1-Mar-1948	USNM				12072828, 107001, 12808
134	NVG-17108D07	***Hewitsoniella migonitis***		M	Papua New Guinea: Lakekamu Basin, Ivimka camp	120 m	-7.70000, 146.80000	R. R. Snelling	18-Nov-1996	LACM				7751324, 111660, 12656
135	NVG-7915	***Aeromachus stigmata shanda***		M	Myanmar: S. Shan State, Kalaw Reservoir			Stephen Kinyon	6-Jun-2002	USNM	NVG170206-10	USNMENT 01321755		10996128, 103431, 12496
136	NVG-7291	***Ampittia dioscorides camertes***		M	Myanmar: S. Shan State, Paya Gyi Gon			Stephen Kinyon	5-Jun-2002	USNM	NVG161007-18			9680830, 90765, 11182
137	NVG-7394	***Astictopterus jama jama***		M	Myanmar: Mandalay Div., Alaungdaw Khattapa Nat. Park	490 m	22.32333, 94.48000	Stephen Kinyon	19-Sep-2001	USNM	NVG161105-67			6219918, 36765, 11898
138	NVG-7381	***Cupitha purrea***		M	Myanmar: Chin State, Alaungdaw Khattapa Nat. Park			Stephen Kinyon	31-Jan-15-Feb-2001	USNM	NVG161105-54			11731143, 103608, 9551
139	NVG-7375	***Taractrocera maevius sagara***		M	Myanmar: Rakhine Div., GwaTwsp Kan Tha Yar			Stephen Kinyon	2-9-May-2003	USNM	NVG161105-48			8394447, 78381, 12655
140	NVG-7783	***Telicota* sp.**		M	Philippines: Palawan				1986	USNM	NVG170205-68	USNMENT 01321623		13288668, 112680, 12190
141	NVG-7910	***Erionota thrax***			USA: HI, Molokai			Dan Robinoff	2-Nov-2005	USNM	NVG170206-95	USNMENT 01321750		14369657, 120294, 12746
142	NVG-7290	***Parnara guttatus***		M	Myanmar: S. Shan State, Kalaw City			Stephen Kinyon	29-May-2002	USNM	NVG161007-17			9647679, 65952, 12293
143	NVG-7389	***Caltoris sirius fusca***		M	Myanmar: S. Shan State, Kalaw Reservoir	1300 m	20.60335, 96.52999	Stephen Kinyon	28-Sep-2001	USNM	NVG161105-62			12767845, 110796, 9903
144	NVG-4767	***Hesperia meskei straton***		M	USA: FL, Levy Co.		29.35675, -82.60340	Nick V. Grishin	27-Sep-2015					13605148, 118482, 11107
145	NVG-4534	***Euphyes dion***		F	USA: TX, Lamar Co.		33.79423, -95.67425	Nick V. Grishin	20-Aug-2015					12319988, 89817, 11118
146	NVG-8381	***Copaeodes aurantiaca***		M	USA: TX, Blanco Co.		30.20033, -98.31613	Nick V. Grishin	31-Mar-2017					14141734, 121578, 12628
147	NVG-9438	***Thymelicus lineola lineola***		M	USA: WY, Park Co.		44.82619, -110.4475	Qian Cong, Jing Zhang & Nick V. Grishin	24-Jul-2017					13857383, 119124, 12626
148	NVG-5158	***Panoquina ocola ocola***		F	USA: TX, Hidalgo Co.		26.25794, -98.64946	Nick V. Grishin	15-Nov-2015					14292886, 118203, 12712
149	NVG-4591	***Calpodes ethlius***		F	USA: TX, Cameron Co.		26.17756, -97.82410	Qian Cong & Nick V. Grishin	9-Sep-2015					14550049, 114348, 11173
150	NVG-8051	***Choranthus vitellius***		M	USVI: St. Croix, St. George Botanical Gardens		17.71666, -64.83333	W. E. Steiner & J. M. Swearingen	9-Feb-1996	USNM	NVG170208-36	USNMENT 01321891		13449553, 117228, 12675
151	NVG-8014	***Anthoptus insignis***		M	Guyana: Acarai Mts., Sipu R.	275 m	1.41833, -58.95333	Steve Fratello et al.	24-Oct-12-Nov-2000	USNM	NVG170207-99	USNMENT 00275220		11704420, 104166, 12408
152	NVG-8059	***Racta chiria***		M	Peru: Madre de Dios, Amazonia Lodge	491 m		Stephen Kinyon	8-Nov-2012	USNM	NVG170208-44	USNMENT 01321899		14278057, 120786, 12175
153	NVG-1769	***Lerema accius***		M	USA: TX, Dallas Co.		32.8621, -96.7305	Qian Cong & Nick V. Grishin	10-Nov-2013	USNM				14616309, 122727, 12698
154	NVG-8007	***Lychnuchus victa***			Ecuador: Napo, Km. 49 Tena-Loreto Rd.	1300 m	-0.71233, -77.74066	Jason P. W. Hall & I. Aldas	18-Mar-2004	USNM	NVG170207-92	USNMENT 01321847		13906685, 116835, 12057
155	NVG-7925	***Carystus phorcus***			Costa Rica: Guanacaste Prov., ACG	305 m	10.77175, -85.43400	Jose Alberto Sanchez	eclosed on 24-Jun-2007	USNM	NVG170207-10	07-SRNP-57133		14631565, 121506, 11690
156	NVG-7958	***Molo mango***			Costa Rica: Alajuela Prov., ACG	415 m	10.90187, -85.27495	Jose Perez	eclosed on 11-Dec-2013	USNM	NVG170207-43	13-SRNP-44273		14077982, 118698, 11679
157	NVG-8008	***Tellona variegata***		M	Brazil: Rondonia, 62 km S Ariquemes	165 m	-10.53333, -62.80000	Brian Harris	14-25-Nov-1993	USNM	NVG170207-93	USNMENT 01321848		6969579, 67551, 11592
158	NVG-7955	***Perichares adela***			Costa Rica: Alajuela Prov., ACG	320 m	11.01227, -85.34929	Duvalier Briceño	eclosed on 4-Feb-2013	USNM	NVG170207-40	13-SRNP-65013		14548671, 120723, 9423
159	NVG-8009	***Lychnuchoides ozias***		M	Brazil: Rio de Janeiro, P. N. de Itatiaia	800 m	-22.45000, -44.61666	Alves & Duarte	1-Sep-1995	USNM	NVG170207-94	USNMENT 01321849		14080967, 117477, 12023
160	NVG-1504	***Megathymus ursus violae***		M	USA: TX, Pecos Co.			Nick V. Grishin	eclosed on 7-Jun-2013	USNM				14982356, 123324, 12795
161	NVG-1670	***Pterourus glaucus glaucus***		M	USA: TX, Denton Co.		33.2536, -97.0434	Nick V. Grishin	4-Aug-2013	USNM				10058844, 86841, 12657

* number of positions from this specimen in the alignment used for phylogeny reconstruction: nuclear genome, Z chromosome, mitogenome. This number indicates completeness of the sequence we obtained.

Collection abbreviations

BMNH	Natural History Museum, London, UK	OM-DZUP	O. H. H. Mielke, Curitiba, Paraná, Brazil, together with DZUP
Brockmann	Research collection of Ernst Brockmann, Lich, Germany	TAMU	Texas A&M University Insect Collection, College Station, TX, USA
CSUC	Colorado State University Collection, Fort Collins, CO, USA	TLS	Texas Lepidoptera Survey, now at McGuire Center for Lepidoptera and Biodiversity, Gainesville, FL, USA
DZUP	Departamento de Zoologia, Universidade Federal do Paraná, Curitiba, Brazil	USNM	National Museum of Natural History, Smithsonian Institution, Washington, DC, USA
LACM	Los Angeles County Museum of Natural History, Los Angeles, CA, USA	ZMHB	Museum für Naturkunde, Berlin, Germany
MNHP	Muséum National d'Histoire Naturelle, Paris, France		

Diagnostic nucleotide characters mapped to the reference genome of *Cecropterus lyciades*

Sequences of nuclear exons with diagnostic characters for the new subfamilies are given. The position used as a character state is highlighted in green. Base pair in this position is the one present in the *C. lyciades* reference genome, and may not correspond to the actual base pair in subfamilies being diagnosed. A reference sequence for the COI barcode region is given at the end. Many positions of the barcode are used as characters, positions are numbered according to this sequence.

Katreinae:

```
>aly528.10.2:G940C | Transcriptional regulator ATRX homolog
ATGCTAGGAAGTAACATAGAAGAGCCACTAAAAGTAAGTGATACAAATAAGCCAACCATGTGCGTTGATACTGTTGT
TACTGAATCTGCACAGACAAAGTCAACAGAAGAAACGGTTACAGAATCGAATGATCCTGAAATTGAAAGTCCTTCAA
AAAGACCAGTAAGAAGAAAGGCAAAAAAAGTATTCCATTATGATGAGGGATCAGATGAAGACCCCTTTGCTAATGTT
GAGCTATCTGATGATGATTTGACAGGGAGAAAGGGTAGATATTATTCAGATGATGAGTATGTTCCAGGCAAAAGAAA
AAAGGGAAAGGCTTATACAGAATCATCAATGTCCGAAAGTGATGAATTTGAAAATACAGAAGAATTAATTTCTAAAA
AACAAATTCATAGAAAAAAAGTGAAGAAATCAGATGATTCACTGATAACATCCCTTTCTAAGAGAAGTAAGAAACTT
GAACGAGATACAATACTAGATATAACATCTACTGCAGCTGACACAACAACTCAAAATGAAGATGATTTGGAAATTAC
TCTTCATTCACCAGATATAACAACAAATGTTAATTTAAATCAATCGCTACATGCATGGGGCACTTCAAATGAATTTG
AGAATTATATAGCAAAGAAAATCCAAGGTAGTAACTTACAAATAAAGAAAGTCTCAGGTACAACACCTCAACAGACT
GCAATAACTCCTCTGGAAATACCAGTGTTGGATCTAAACGAACCTAAGAAAACTGTAGAAATGTGTACTCAGACAAA
TGCAGTTAGCACTAAATCAATTGAAGTACAGACAACTTCTCCACATGAAAGTAAAATGAAAACGAAAGTTGCCTTAA
CTGCAGATCAGTCGGAAAAAGCCTGTGAGTTTTTAAAAGGCATTGTAAAAACTACTTCCGATTTAGGACAGTTAATG
ATACAAAAATCTGAAGACTTTATCCAAAAGAAAATAAATACACGGAATGTTACTGACACAACTAAGATGGATTACTG
TGTTCAGAAATCATTTTTATTGTTCAAATTGGCTAAGCATAATTTAATACAAATGGAAGAAGATTTAGCTACTGAAT
ATGAGCGATTTCTTAGAACTAATAATATGTTACAATATCAGGAATCACCTAAGTGTATAACAGCAACTCCAAAACCG
GTAAGTAGTGACAGTGACTGTGAAATAGTGGAGGAGCCAATTCCTACCAATACAAAAGAAAAGTCTAAGTTTAATCT
GAAAACTGTCTTCCTAAACAAAGAACTTTCTATAAAAATTGCAAAAAAGTCTACTGAAGAACCACAGATAAATAAAG
AAAAGTTGAATATAAAAGGCAGGCATACTGTCTGGATTAATGATTCCATAATGGTAAAAAAAGTCAAGCCTACACAG
TCTTTTTTAGCCCAGGACAGCCGAAATAAAAAACCTCCTGATTCTTATATAACAGCAGAAATG

>aly925.27.5:A3610T | Methyl-CpG-binding domain protein 5
ACGCAGTCGCACGAACTGGAGGTTAAACGAACGGCTTCACCAGGTACAGCGTCTCGCGGAAACATGCAGCCGGGACT
GCCCGCGTACCCGCCTCTACAACAAAACATGCATACAAGCGTTATAACTCAAGAGACTGAGATTAAACAGGAAACGC
AAATACAAAGAAGCTCACATTACCTCACAGTCAATAGCGGCTGGATAACGCACCATCAAGAACATTTGGAAGCCGTT
CAAAAACAGAACCAACCAACAGGCGGCCACTTGCACCCTTCTTTAAATGTTGGTTTGCCAGTATTGGGTACTAATGG
TCAAATTATTGGTGTGAATACAGGATATAACAAACAGGTAAACAACATAAAAAATCTTATGGGTGTAACGCCACAAT
CTATACTAGAACAAGAGCATAAAGAAGATAAAAGAGACATTCAGGCAGCTACTCAGTCCATGAAAACGTCGAAAGAA
GATGCAGCTTTCTGCGGTCTAAATCAAGCTTTAACTCCAGAAGTCATGCAAAAAATAAATGAACAGCAGCAACTTTT
TATACAGCAAAATCAGAAACAGATAGAAGTTATGAAAGGTTTTAAGACGCAACTGGCCGAGACCGTGAATCTTAATA
AAACTCAGGTACAAAATCAGTATGTTCCGCAAGGCCAGATGATGATACAAAATGGGTCCGTAGTAAAAACAAATTCA
GCAAAAACACCTCCGTGGCAAGTAAAGCGGAATGATTCCAACGCCTTGACGAGTCCTAGTCCTAAACCTCAAAAACA
AGACAACGCTGATTATGATGACTCAAGAATTAGCGTAGAGGAGGCATCGGCCGTGTTTAATTCTTATCCGAATAACG
ATCCTTCGATGAATCCTGCCATGTGTTCTCGAGTGCCGCCATTACCGCAACATTATAATATAATCGGCCAGCAGTGG
CCGGGAATTGAACAAACCAAAAAGAAAACTAAATCTCACAGTAAAAACTCCAAAAAGAAAGCAAGTCCGACGGAAAA
TAAAGTTATGTTATACGGCAACAATGGCATTAAATATATGCAGGATCCTAAAAACAAAAACTCTGATGAAGTCCTAT
GTAATAATGTACCGTCATTTATGGAGGATCCCAGCGGATATTTAGCACAACAAACTGCTTTACTGAACAATACTATT
TCCAGACAAGTAGGTATAAATTCTTCCTTCGAAGGTCATCAATATGAAAACTCTAGCAGCTCATATTGTGTCCAACA
TCAAAATGAAAAGCAATTGAAAAATATTAACAAAACTTCTGAAACGGTCAATGTTTATAGAAGTAATGTAGTGCCAA
ATTCTTCACCCTCACCGAACAACACGCCAGACAGCAGTTTAATACCAGATAAAAATATGGATTCCGCGTCACGATGC
TGTAAGGGTTGCAATGTTTCGTATAAGGGAAATTGTACAGATTTTTCGCATAACCAAAATAGAACTAAATACTTAAA
GCAGGGCGTATACGGAATGGAATTGGATGGTAATCCGAGCACATCGAGTCCTAAACATGGTTTCGACGATAATCCTG
TAACATCGTCTACATATATCGAACGCAATGTAATGATGAATGATAATTGCGGACCTATTCAAGCCGGCATAGTCAGT
ACTAGTAATGTATCACCCACAGAGACCTTACAACCGCCGGAACCGTCGCCCACTTTGTCTAATAGTTCCCGGAACAC
TGACACACCTCATAGCAGCGGAAGTAACAACTCCCAAATGCAAAATAATTGTACATTCCCAATGCCGAGTCCAGCAT
```

ACTCTAACCCAGGTTCCTGTCGGGATAGCCTCAGCAGCAACCCATCTACTCCAAACAGTAGCCAACCGTACCCTTAC
AACTCGCCAGGGCCTCCTACCAATCAAGCCATGTCTGGGAACCAGATGTTGCATTTCATATCAAACCATAATATTAA
CAAGAATCTTATAGAAGTAGAAAACATATCAATAGTTGGCATGAAGCCTGGTTCACAAAATCTTCAAGAAATGTTTA
GATTAGAGTCTAAGAAAAGGATGGAAAATGCAATGCCCGGCTATTGCCCTCCGCCTCATATGGGAGGCGGACCAGCT
CATTCATTAATACAAGCTTGTTACGTGCAGACCTTCGTAACGACTATGGCGAGTGGATTTTCGGTCACAAGAGACAC
AGTCACGTCAGTATTAGCTGGTAAAGCTAATACTGCAACCACTTCTATCAATGCTTCCCAAGCCAATTTCATTAGAC
CACCGCCACCTCCAAGTGTTAATTTAGCTACGACTTACGCCATACCGAACAACCAGCCTGATCCATTCATAAATTCT
GTCAACTTACCGACCAGCTATCCGCTCCATATAGCTGGAACGACTGCGCAGAATATGATATCTAAATCGCCTCTAGA
GATGGTGCAGAATGTTATTAGCAGTATGCCTTTGAAGCCAGAAACAAGTAATACTCAGTCTACGGTTTTATCACAAA
TGGGCAGAAGAAGTTCTCCTGGCCAAATTGTTATTTCGTCAACGGGTCAAATACTCGTGTCAAGCAATCAAATGCCA
CCGCCGCCTCCCAAAAATACTACTACTATGTCAGTATTGAGTTCCAATGCTGTAACAAATGTTACCACGTCTGTAAC
ACAAGTGGTACCGGCAGCCAATCACATCCAGCCACTTGTGAATCAACCTACAGTAGTTGTTAATACATTACAAGCTC
CGTTTGTATTGCAGCAGTCGATGATACCCGTAGACGGGCAAGTTATGCAACAAAATCAAGTTATTCCACAATTAGTT
ACGGGAGGCATCGTTTCTGGTCAGACTGGCTCTGAGACTACGCGGCAAATTGAGGTCAAAAATGGACAAAACTTCGT
ACAAGGTGTAGCTATGCTTTCACCGGAGAGCTTAAAGAAACGCAGTAAGAAGAAGAAAAACCAAGCGGCAAATATTA
CGAACGTTTTACAAATAACAGCACCGCAACAAAATCCAAATAACATAATGGTGCATCATTCTTCACCGCAACATAAT
TCTAGTCCACAGTTTTCTCCCAGAGGCTTTCAGTTATCGCCAACCAATAATATATCACCGACACCGATGTTGCAAGC
TCTGACCATTATGCCTGGTAAGTCCGGTACACCAGCGCACATCGTTATGAACGGGCAAGGAAACTCCAGTAACTTTG
GATCTCAACAAATCATTGCTAATACGTCACCATCACAACAAATAAATCTGTTACAACCCGTGAATTTGATCAACAAT
GCGAACAATGTTATGTCGAATTTCCCTGCTTTCCAGCAGTTCATTGTGCCAAATCTTAGCGGGATGGTCATGACCGC
CGATGGAACGGCCATTATTCAGGACAATTCAACTGGAATGCCAATGCAATTACAAATACAAACGGTCAATGGACAAA
ATGTTTTAACCCCTGTACAGAATTCTGGCGTTTTTACTGCAGGTGGAAACAGTGGTGTAGTAATCAGGGCTCAGAAC
CAACAAGGAAAAATAATCCAGTCGCCGCATAGTCCGGGGGCGCAGTTTTTGTCACCGAATAGTCAAGTTATGATGAA
CAGTCCGAATTTCAACGGACAGCTGAGTCCCCTTCTGGCGAACTTAAGTCCGACAAATGTGACTTTCAATACTAGTC
CTCACCAGGTTCGATCCGGGAACGTCCAAGCGCAGGAGTTCATACAGACTAATCAGATGGGGCAAACATTGATGGTG
CCTCTGTCTCCTAAACAGGCATCAATAGGTTCGGCGAACAACAGCCGAAACTCCACATTCGTGCAGCAGAATACGAC
AATAGTGCAGCAACAGACGACTTTAGTGTCTAACAACGCTCAGTTGCCATCAATGGCGAATCTTCAGGGTAACTCAC
GGCTGAGCGGGGAGTCGAACGTGATATTGGCTCAGAAACAAATCGCGCAAAAGGGTCGTAATTTCGTGCAAATCGAG
GCCGGACCAGCGGAGAAAGAAGAGAGACGCGCATTCGGGCGACAAGTGAGCGAGAGTGAGACGAAAGATGACTACGG
GAAGTTTACGGGTGAAGCGGGCACGGCGCACGCGCGACATACTGACGGCGAGTACGGGAAGCTTACGGGTGCAGCAC
ACGTTCGACACACGGATGGAGATTACGGGAAATTGACGGGCGCGGCACACGTTCGACACACGGACGGCGAGTATGGT
AAGCTGACGGGTGCGGCACACGTTCGACACACGGACGGTGACTACGGAAAGCTGACGGGCGCGGCACACGTTCGACA
TTCAGTGTCGACGCAGACTCGCAAGTCGCCGGGCGCGCCGGCCGGCAGCCCGCCGGACACGACAACACACAGCCCGT
TGGGCGTCGACGCTGAGCCCAGATGTATGTCCACGCAGGGGAACTACGCAGATACAACTACTGAGTCCCCGGAGCCC
GCGGATAACAGCTCATCG

>aly84.77.5:T1651G | Zinc finger protein Xfin
GGTGCAGACAGTGATAAAGATGACGAGGAAGAAGAAGAAGACGACGAAGACGGGAATGAATCCCAAAATTTTTCACC
CATAAACATTTCTAACAGATCCGGGATTCCCAATATGCCTGAAGTTTCCATAACTGTCATGCGTCCTACAGGTGAAA
CTTTACACTCTCGTCAAGGTATCCAACAACTTGCTTCTAAAGATTGCCTCGTATGCGGTCGATCCTACCAATACTCC
CATAATGCAAGACGTCATGAACTCACCTCACACGGCTTCGATAGATATACAAATAAAATCACACACAAAAAAACTCC
TTCACACATGCAACCAAAACTTCGACCTAATCCATTCAATCCTAAAGCTCGGCTTATGCCTAATCCTATCAGCCATA
AAATGCAACTTATCCCGAAGGGTATTTCAACAAAAATCATAACCCCAAAAACGAGTGGCCCACCTAAACCGATCCCT
ATCAAAACTATAAAACCGCCACAGCATATTTTGCCATATCCATTGCGTATCAAAGCACTAAAGGATTTACAAATAAA
AAAGAAAGAACCTCAAATTTTGAAAACACTCCTGACAACTAAGCCTGAAGTTTTAATATCTGAACCAGAAATAATTA
ATTCGGGTCCTGAAAGTCCGGAAACATTGATATCGGAGCCCGAAATAGCATCGTTTCAAGTAGAAGCTATTCTGTCG
GAACCAGATACTTTTGTGAACCAACATGATGATGAAGGTGATGGAGAATCGCATCAATACAAAACAAATAATCATGG
CCAAAACTATGATACTGTAGAAATGGACACTGATAATGAAATAGAAATCGCTCGTCAGCAGGAAAACGAAGGTGATG
ATGGTCAAGTGGGAAGTAAAGATGATACTGAAAATGATAAGCATATAGTGGAAAGCGACGAAAATTATAAGCAAGTG
CCAACAGAAGATGGTGAAATGCATACTGATGGCGATGTTGATGTCAGTAAAATTAACCACCAAATCAAAGAAGAAAA
GGACCATGAGGAACAAAATCATGATGACAATTATGAAGATGATGATGATGAGTTACCACCAAGCTTGGCTCCTGTTG
TAGAAATTAATGAAGATATGCAGGCAAATTCTTACAATAGCGAGATGAATGAAGAGGAAGAGGAAGAAGACTTAGAA
TCAGCAGATGCTAATGATACAATAGACGGTGAAGAAAATGCGAAAGATCTAGATACCGATAAGATATATGTTACAAA
AACTCAACGAGACTTTATAATCAAGTATCGTGATATAATTGAGCAGATTAATACCAAGCGATGTCTTTGCTGTAAAA
GGGAACATCCCCGCAGAAAAGCGGTCATTCAGCATTTACAAAAGAATGGCCATAAAGTACCCAAACATACTTGCTAC
AATTGCGTTGTCACATTTACTCATATTGGAGCTTTACTCAGTCACATGAGGTCAAACACTTGCACTGATTTGTGGAA
AGTAATTTATAATGAAAATGGCATTACGGAAGATCTTGTTTTAGAAGATGAAGAACCAAAAGATCCTAAAATTCAGC

CCAAAGATATTTTTAACGCTAGATCATATGCATGCAAGTTATGTCCAGCTAAATTTCAGTTGAAACAGTTTATAATG
AAACATGTGTTGGATGTTCACGAAGATGGCCAATCTAAAGTAACGTTGGCATGTGTCCATTGTGGATCGAGATTCAA
AGATAAAGGTTTGTGGAAGAAACATATACGAAACGGAGAATGCACAGTTTATATTTCGTGTGACCTATGTTCAGAGA
AGTTTGTTAACATGCAAGATTTTAGCGATCACGCTCTGGCTGTTCATGCTGGCAGCTGCGATCTATCTGATAGTCAG
AGTAAATGTGTGGACGGAAGACCAACGGACTGTCCTATATGCGGCAAGAAAAATAGCAGCTATCCGAATTTGGTAAA
ACACTTGAAGATTATTCATAATGAGGAAAAGCCTCATTACTGCCAGCACTGTGACTCCAAATACGAACAAGCTGCCG
AACTGAACAAACACATTTATATGGAGCATTCAGACAGATCTTTAGGCGTCCAGATTTCAACGGAGCCCGATATGTCG
CTCGTCAAAGAAGAAGCGGAAGAATATCATTATTCTTGTACGGAATGCAATGCCATTTTCGAAACCGTGGATGCATG
GACCGATCATCAAGTAGCTGAACATAATCAGGTTGCTCATCACTGTGACCAATGCGAAAAGAAATTTTTGCGGCCAT
CTGAGTTGGCTGAGCATAAAAATACACATTTGCGGGTGAAATTTTACCCTTGTAATGTCTGTTCAAATTCTTACAGC
ACCCCTCAGAAGCTTTCTGAACACGTTCAACAAGCTCACCCTGGGTCCGGTGGATACAGTTCATTGGATGCAGAATT
CTTTTGTGACATATGTATCAGATCATTCAAAAGCCGCCAGGCGTATTCTAATCATATGCGTATACATTCTAAAGTAC
CTACTACTAATAGGAAGCCTGGAGATCCTAAAGGGTTTTCACCACAAATTATTGGAAAACCTATTCGACACTTTTCC
ATGGTTCAACCAAGCTTTTTGTCATTTAAGCCAAATTGTAATGTTCCTAATGCTCCTTACTCATGTGATATTTGTGG
CAAAGGGTTTATGCATAAGAAAAATATATGGAAACATAAGAAAGTGCTGCACGCTGATCTGATTAATGACAGAAACG
ACAGTGAAGAGAATACTATGCAAGCATCTACCGAAGAAGATGACTATAACGTAGATGAAAATGGCTCCATTTTGTCA
ACGCCCCAATTCAACAGTTTCAACTTTACAAATACGACTAATAATATGCAGCAGCAGCAGCAACAGCAGCCCACACA
AGACCCGATGCCGTTCTCTTGTGAATTATGTTACAAACGATTCCCTCTTAGAACTAGTTTGTGGAAACACAAACGCG
CTAAGCATGGAATAATTAACCCTGGTTCTACTAAATCGTCTGACCAAGTAACACCTAGCGAGTCGACTGGTCGGTCG
AATTGTACTATATGCGAGATAAGTTTCTCCGACAAGAGGTCGTATTATCGTCACAGGAAAAATGTTCATAAATCAAC
TGTACAGATGTGTAAGATCTGCGGTAAACCGCTAAGTTCCACGGGTGAACTTTATGAGCATTTGAGGGCCGCTCACG
CACAAGAATTGCTTGGTTATAATGCAAGCCAAGGTACTAGCAAAGCGCAAGATATGAGTCAAGAAATGGACCCAGAT
TACGAACAGGATCAGGATACCATAGATCCTAATGCTGACTATCAAGCACGATACCCATGCGATACTTGTGGAAAGCA
ATTTGTTGGACTCCTCGCATTGCAAAATCATCAATGCATTAATCAGATGCAACCGCAGCCGCAAACATTTGACTGTG
AGATTTGCCATAAAAGCTACACATCTATTGCTGCTCTTAAGAGCCACCGCGGTTGGCATTTGCGCTCACCTGACGGT
AAAGCTGCTGCAAATAATTCCGGCCTGTGGATGCCACAGCGCAAAGTTACAAGTAAAGTAAGTAAGCATGAAGTAGT
TGATCCCTCACAATTGGCGAAAGTTACGCACACTCCCCCAAGCGTGGTAGCGAAGCGGAGGTTACCTCCAGAAGTAG
AAGTTACCGTGGTCAATCCGAACAAAAAATTGCGATCGGATGATTCCGTTGAAATTGATCAACAGAACAATTCATCT
TCAGGAATCGAAGACAGGTATTGTTCTATATGTGATAAGGAATTTACGAAACGGGCGGCATTCCAACGCCATATGGA
TGAGGTCCATCAACCAAATTCAGTGTTCTGTCCTGTTTGCGACAAAAGTTTTACTCGGAAATCGACGTTGATTGTTC
ACATGAAAAAGCATTACGATGCCGGAGAGGGTAGTTCTACAGGTACAGCTCAGATGGATGACGATGCTCACGTATGT
GACGTCTGTGAATCACAGTTCGAAACTGTTACCGAGTTGAACGCTCACCGTATTGAGCAACACGGTGAAGGGGATGG
CGATGGAGAAGAAACGGGCGAGTCAGAGGAGGAGGCAGTTGATGTAGAGCAACCTCCAGGCGAATTCACTTGTAACC
AATGTGGCGATGGAGTGGCGACACCTCGTGATTTAATTGCGCACCGCACCATGCACTCTACACCGACTAAGTTCTTC
TGCAACATCTGCAAAGTTTACTTTGCAAGAGCTTTGGATCTCTCGTCCCACACCAGGGCGAGACACTCAGACAACGA
CAAGGTTTCCTACCCATGTGCCATGTGCGACCGCTCCTATATGAATAAGAAGAGTTTGCAGCGTCACATTGAAATGG
CACAC

>aly595.14.2:G184C | Endoplasmic reticulum lectin 1
AATAAAGAAACACTAAAAGTACTAACACATCATAAGGAGAAATATGAGTGTCAATTTCCAGAGCTATATTCCAAGGA
GTCTTCTGGAACTGAGGAATATGATGGCCCCTCCCCAATACGTCTCTTGCAACCACTGTTCTCTCAGAAGATATGCT
CCTACAGGCTTGAGAGTTACTGGAGCTATGAGGTGTGCCATGGAAGATATGTAAGGCAGTATCATGAAGAAAGGGAT

>aly2284.22.2:G967C | Kinetochore-associated protein 1
GTGCATATATGCTGTGCTGTATCGTCTCACTGTCAAGACCAGCAAAACTTCGATAGCACTATTATTTCTGCAGCACC
ACTATCTACACAAGAGTCACTTAAGTTGCAGTTGAAGTCAATGGTTGAAGAAGCCATTCAAGCCATGAAACCGCAGG
TTGAACAATTTCAAACTCTTTTCGACAGTATAACTGATTATACTTTCTTGATCAATATTGCAACTAAACTCTGTAAG
CACAATTTAACATATAAATCTTTATTGTATCAACTCCAAAAGAAAGTATTTAGTACTGATGATAATAATCTCATGAA
ATTATTTTCTGATATTATGATAAAAATCGATTTATTAGAATATATATGTTTTAGGGGGAATAATTTATATGCGACTG
TGAATTTATTCGACTTAAATTTTATTGAACTTTGTGTAACATTTATATCCAAAAGCGATTTAGATTTGGCATCTATT
TGCTGGTTAAAATATTCTGAAATGAAACTTACTATCAAGCCTGATGATATTGTAAATATTTTAAATGCTATACCTGT
AAATATTAAAATGGGAGGCCTAATAATCTGGCTCCGAAATTATATCCCCCCACTTTTGGATGTAAACCCATTTTATA
TAGATTTATTTGTAAAGTGGACAACTGACAGAGTGTTGTTGTTAGAGCAATCCACTTACTGGCCGAAAATAGGTTTG
AAATTTATTGAAGATATCGCTGGTGTTTTGGAAAAGTCACTAAAAACCATATCATTAAGACCAATTTCTATTGATGA
TTTAGATGTTCTTAAAGATCATATAAATTATGTGATGGAACTAAAAGAAAAACACAAAATTAATATGCTTCTGAGTG
AACTGAGTTCTCAAAGCCCAATAGAAGTTGCACTTATCATGTTGCGTCGTTGTTATACTGAAGATCTTGAGATATTT
TTACAAGGAAGTTTGCCTTATTATGCAGCTCGATATCAAATCGAATTGGATGACACTCTTCGCTCATTTATAGAAAG

TGAAGCCGCTAGTAGTGGAGGATGTGTAGATGGTCAGAGATTACAAATATTGCTCAATGCCTTTCGCTCACCCAATA
ATAAACTTGAGTGTCTTCTTCAAGTTTTAAAAGTATTAGAGGTACCATGGAATGACACTGTACTCAAAATAGCCACT
GATGCTGCAGCATCCGCTAATACAGATTTTACGGTAACAGATGGTGATCGCGCTATTGCTCAAGAAATTTATATGGA
ATTAAACTATGCCAGAATCAAAGTTATTTTAAAGAAATACAATTTTCCAATTACCTTTACTAATTACGTCACAGTCA
TTCATAAAATAATAAACGCTCCAACTGTCGATTTACAGGATTTAAAGGTAATTACTACTGTAGTATCTGCATACTCC
GATTACGCTCACCTGTTGTACATCGATAAATGCTTGCGAGATTGCGATACAAGTGTTGCATTGGACTATTTTAAAAA
TTTACCCAATAGTAATAGAAAAGTACTATTGCTGGCAATAGTAAACAAATACGAGCAAATCATTAACAGGAAAAGAA
AGGATTCTACTACAGAAAGGAATTATTTAGACTTGTTAAAAGGAATCAAATGTCTTGATGACAAAATTATCACAGAG
ATTGAAAATTTGTATCATTTAAAAAACTCATACAATATAACATTCAGTTTAAATAACATATGTAAGGAAAAAGTTCG
AAATGACGAAGCAAATAATTGGGAACGGCAAGTTGGAACTACCGCCAGTTCTGGGCGAGGACGATGTGTAAATAAAT
TGTTACGCATAAACTTTTCTCGTGATTCAAGTCTATTAACGTTATTGCGTAAAATATCAACGAGCCCCGAGGTACGT
GATTTTGTTGAACGTTTTGTAGTATCAAATCAAGACAATTTTTCCATCTTATTGCAATTTAAAGATGGTGGGAACTC
CTCCCTTTTATTGGAAGCTTGTAAAGTCATGTCACAATTAATATTTAATTGCGTAGAAGAGTATTTACATTACTTGA
TTGAGCGTTTAGCAATCTTAAATTGTTTCGTTAATTCAAATATAACGATGACGAATCTATCCGTGGTATGGAAATTT
CACTACCTATTTCTACCTATGTCATCAGTGCCTGCATTGAATGATTTGATTGACTTCTACATAAATATTAAAAGTAT
GGAAATGTGCAATATTACAAATAAAAGTGATTTCATTCCGATTCGAATGGCAGGTAATATAATGTCTCGAAGTATTA
AAAATAATTCTAATTTATCCGATGATTTCCTCAAGATGAGAAACATAATTTGTAGAAAACTTTTACCTAAAGTTGTT
GCCGCACAAGAACTTGATCACATTCTTTTCACCAGCGTACTTTTAACGCTTTGCGATGTTGAAGAGACAAGTGATAA
TTCATGGATTTTGGATATATTAAGAGGGCAATCAGATTCATTAAATCCAGTTGTTATGCATTACCTCTCTTCACCAT
TGATTCGACGTACTTTTGATTTGTACAGTCTCATTCCAGGCAGTAACATGTCTTATCCACCACAATACGTTCTCAAA
AATAAATTCAATATTAACCTAGCTGACATAGCATTACCTGAAAACACAGAAGAAACTTGGGATGTGAAAATTTTATT
ATTTTATATTTTAAGACACTTCCCAAATACCTCATTTGAACGACTCTCAGATCTATGTCACACATTAAATGTCTCGC
TAAACTATGGATTGTCCTTACTATTGATTTCCATATTGAGTAACTTTGATTTGAAATACAAAATAAATGTAGACAAC
TTGGGATGTCGACAAATTTTGCTGGAAAACGATGAAAATCAATTATTGTCCACGTGTTTAACAATTTGGGAAAGTAT
TAAAAATAAAGATTTTATAAAAGATATACTTGGTGACTTTTGGAAGAATGGTGAAGTAACATTACACGGATGTTTAG
TTTCGATAAATCCATACCATTACGAAGTATATTTGTGCATATATTATTTAATATTTAACTCAACGCCCGAGTTACGA
AATACGAAAGAATATTTCTTATTAAACTTTTTAAAAGATTATAAAAGAAAAAGTTCTCCAAGGCAATATGAGTTTGA
ACTATTTTCGGTAAAGGGTATGTTTCCTGAAATAGGCCACTATCGCCTACCATTTCATCTATTTCTGAGAGACGATA
TGTGGTCCAATTTAAAGTCTGAAATAACATTGGAAACGTATGAATATTGGCTACCTGTTGTTCCTTTATTGTCACTT
GATAGTGATTTGCAGACTGCAAAAGATATGATATGCAGCAATGCTGTGAAACAAACTATGACTTGTCGAAAACGTCA
AGATTCTACAGAGTTAGATCCAAAAGAGCGGGAACCATGGCGCTTAATATCACGAGAAGAACCCCTATTGCGGACGG
CGCATCGATGTGTAAAACATATCGCAAATATGGAATGGGCTGGTGCATGTTTATTTTACGTGTTACAAGGTTGCGCT
CGTGGTGCTGATCAAGTTGCTGCGGCCCAGCTATGTTATCAGTTTTCACAACGTTGGGCTTCGGTTCAACCGGGGAA
TCGAGCTGTTCAACAAATGGCACGTCTACATTCCTCTTTATCGACCCGTCATGCTCTTCATAAAATAGATTGGGCTC
GTGAAGAATTAATTCGTCTGTCTACTGAACCTGCACAACTGATACAAGCCCTATACCTTCATCCTCAGTTTGTTGAC
AAAGTAACACGGCATGATGTAAATAGAGCAGCAAACGAAATAGCTGACAAAAATAACGTCAATATAAGTTCGATCAG
AATACAAATTCTTGAAAGTATTCTCGATAAAACTACAAAAGAAAATAAACATTCGGTTGGCCTAGACACGAAGGACT
TGATAACTGCTAAATATATATTAAAAGCAACTTGTCCGAAGATGGGAGCTATTTATCTATCTCGAATAGCATTTGAC
GATGATAGTGACTTTAATAAATGTAAAAAATTAAGAGCTCTGCAGTGTTTAATGAGCGTTATAGAGCCTGAAACTGC
TGTGAAAGTTACTAACCGAGAAAGAGAAGCACTTTGGGTTTCACTTTTGGACTTGCTTTTTATTGTTAACCTTGAGA
AAATTGATATGCCTTGGATAGTAGCTACATTTTTACAAGATAAAATACTTGCTATCAACCAGTTGATTCAAGTCAAT
AATATTAATGTGGAAGGATTAAAAGTAGCAGCTAAATTGGCCCATATGTATGGTAACATAAAAGTTATACGTGACCT
TATACCGCGTTTGTTGCGTACTGCATTATACGAGGAAATGATTCCATTATTACTACAACTACTTTATCCGATCGATA
ACGTCATATGTACAGCTTGGCGCGCAATAATCTTAACCCCATTCCAACGAGCTGATTACCCTATAACAGAACGCCAA
AAGGCTAAGTGTCTCTCCGCTTTAAATCTATTGCCAGTGTGTCCGGTAATAAACGACGATGATTTGATTGAGATTTG
GAAAAATTGCGTCAGGTGTAAATGTTATGGTTTAGGTTGTTTAGTACTTCCTTATATAACGGCACAAAGAAGACAGT
CGCTAACGGAACTTCATAAAATAGACAAAAGAAATTTGATAATAAGTTTAAAAAATTTGCAAACAGATACGTATTTG
ACATCGGGTGCTATGTATGTTTTAGAAAATATGAGTCAAAAAGTTTATAGA

Chamundinae:
>aly528.10.2:A631C | Transcriptional regulator ATRX homolog
ATGCTAGGAAGTAACATAGAAGAGCCACTAAAAGTAAGTGATACAAATAAGCCAACCATGTGCGTTGATACTGTTGT
TACTGAATCTGCACAGACAAAGTCAACAGAAGAAACGGTTACAGAATCGAATGATCCTGAAATTGAAAGTCCTTCAA
AAAGACCAGTAAGAAGAAAGGCAAAAAAAGTATTCCATTATGATGAGGGATCAGATGAAGACCCCTTTGCTAATGTT
GAGCTATCTGATGATGATTTGACAGGGAGAAAGGGTAGATATTATTCAGATGATGAGTATGTTCCAGGCAAAAGAAA

AAAGGGAAAGGCTTATACAGAATCATCAATGTCCGAAAGTGATGAATTTGAAAATACAGAAGAATTAATTTCTAAAA
AACAAATTCATAGAAAAAAAGTGAAGAAATCAGATGATTCACTGATAACATCCCTTTCTAAGAGAAGTAAGAAACTT
GAACGAGATACAATACTAGATATAACATCTACTGCAGCTGACACAACAACTCAAAATGAAGATGATTTGGAAATTAC
TCTTCATTCACCAGATATAACAACAAATGTTAATTTAAATCAATCGCTACATGCATGGGGCACTTCAAATGAATTTG
AGAATTATATAGCAAAGAAAATCCAAGGTAGTAACTTACAAATAAAGAAAGTCTCAGGTACAACACCTCAACAGACT
GCAATAACTCCTCTGGAAATACCAGTGTTGGATCTAAACGAACCTAAGAAAACTGTAGAAATGTGTACTCAGACAAA
TGCAGTTAGCACTAAATCAATTGAAGTACAGACAACTTCTCCACATGAAAGTAAAATGAAAACGAAAGTTGCCTTAA
CTGCAGATCAGTCGGAAAAAGCCTGTGAGTTTTTAAAAGGCATTGTAAAAACTACTTCCGATTTAGGACAGTTAATG
ATACAAAAATCTGAAGACTTTATCCAAAAGAAAATAAATACACGGAATGTTACTGACACAACTAAGATGGATTACTG
TGTTCAGAAATCATTTTTATTGTTCAAATTGGCTAAGCATAATTTAATACAAATGGAAGAAGATTTAGCTACTGAAT
ATGAGCGATTTCTTAGAACTAATAATATGTTACAATATCAGGAATCACCTAAGTGTATAACAGCAACTCCAAAACCG
GTAAGTAGTGACAGTGACTGTGAAATAGTGGAGGAGCCAATTCCTACCAATACAAAAGAAAAGTCTAAGTTTAATCT
GAAAACTGTCTTCCTAAACAAAGAACTTTCTATAAAAATTGCAAAAAAGTCTACTGAAGAACCACAGATAAATAAAG
AAAAGTTGAATATAAAAGGCAGGCATACTGTCTGGATTAATGATTCCATAATGGTAAAAAAAGTCAAGCCTACACAG
TCTTTTTTAGCCCAGGACAGCCGAAATAAAAAACCTCCTGATTCTTATATAACAGCAGAAATG

>aly3277.11.2:A1726G | DNA-directed RNA polymerase I subunit RPA1
GGCCTGAACATGGATTACAAGTCTGGTTTCACGTCGGCTCTATCGGGGTTGACCTCCGTTGACCCCGACTCTATACA
GTTCCTCATGTTCAGCGATGAAGATATAAGAAATCTTAGTGTTACTAAAATAACAAATACTATCTCATTCGATACGA
TGGGAAACCCTAGTAAAGGTGGGTTGTATGATCCAGCTCTAGGACCTATTAGGGATAGAAATGATTTTTGTTCTACT
TGTTCCAACTCCTTACTCCACTGTCCTGGGCACTTCGGTCATATAGAGCTTCCGTTGGTTGTTGTCAACCCACTCTT
CGTGAAGAATATTTATACCCTATTCCGTATCAGTTGCCTAAAATGCTTCAAAATCCAAATGGACGACAGAACAAAGT
TCCTACTAAAGTTGCAGCTCCAACTGCTTGATGCTGGTCATATAACAGCGGCTTTGGACTTAGCGCACTTCATTGGT
GATGTCAAAGAGTTTAAAAGCGAGACATCGCCTGAAAAAATGAATAAAACGATACGAAAATATCAGAAATTAATCAA
AAAGTCAGACCCAGTCGTAGATGTGTTCCACAACAAAAATACAGATAAATTGAGAAATCAAGCAATCACTAACGTTT
TCAAAGCCGTCAACATATCTAAGACATGCCTATACTGCAGAAGTAAACTCATTAAAGTTACTACATCTGACAACAAA
ATTATGTACAATCTTAGTGCAGAAAGTGGAGGTAAAGGAATTAAAATATTGATGCCAGATGAAATCCAACGTTATTG
TAAAGCAATCGCTCAGAATGATGAAGACATTTTGTCTCAATGTGTCCCTATTCTCAAGCATTCCAAAACAAATCCAA
TAACAGATATACTATTCATGAAGTTAGTGCCAGTGCTTCCACCTTGTGTAAGACCATGTAATGTATTACAAGGAGAG
TTAGTTGAACATCCACAAACAAATGTCTATAAAGGTATCCTTCAATCTGCCTTTTCTGCAAGAGCTGTGTTACAGGT
TTTATCTGGATCAGACCAACAAAAGGCGATTGATGGTCTTGACCAATTGCCCAGACAGGCTTATGAAAGTGTACAAG
GTAAAACTCCAGCTGAAAAACTTCATACAATATGGCAAGATTTACAAAAACATATAAACTACATACTAAACTGTGAA
GGACAAGGTGTATCCAGTCAAGGGCTAAAACAGATTCTGGAAAAGAAAACTGGTATTATTAGGATGCATATGATGGG
AAAAAGAGTTAACTTTGCTGCTCGGTCAGTCATCACACCAGATCCAAATGTGGATATTGATGAAATTGGTATCCCCG
ATGCATTTGCCACAAAACTAACCTACCCAGTACCAGTGACCGAATGGAATGTTGATGAATTGCGAAAAATGGTCATA
AACGGACCAAACAAACATCCGGGTGCTGAGAAGCTTGAAATCAAAAATGGACGAGTAATAAGAATACCTCCAGATTC
TATTCAAAAAAGAAAATCACTAGCCAAGAGGCTACTAACACCTGATGAGTACAAATCTTCTGGTTTAAAAATTGTTC
ACCGACATTTAGTTAATGGTGATGTGCTTATTTTAAATCGTCAGCCCTCCCTTCACAAACCTAGTATGATGGCTCAC
CGGGCAAGAATTCTTAAGGGCGAAAAAACATTAAGATTGCATTATGCCAACTGTAAATCTTACAATGCAGATTTTGA
TGGTGATGAAATGAATGCCCACTTTCCCCAAAATGAAATTGCTAGAAGTGAAGCATACAATATAATGTCAGTTACAA
AACAGTACCTCGTACCAAAGGATGGCACACCATTGAGTGGTTTGATTCAAGATCATGTGATATCGGGAGTAAAAATG
TCAATCAGAGGAGCGTTCTTTACTAAAAGCGATTACCAACAATTAGTCTTCCAAGCTTTATCTAATCATAAAGGTGA
AATCAAACTTTTGCCTCCAACAATTTTAAAACCTATAATGTTATGGTCTGGTAAGCAAATTCTTTCTACAATTATAA
TCAATACCATTCCTAAGGGAAAACCATGTCTCTCATTAGATGGAAAAGCTAAAATCAGTGCAAAAGCTTGGCAAAAG
GAACCAGCAAGAAATTGGAAAGCTGGTGGAACTCCTTTCACTAATCCAAATGCGATGTCAGAAGCAGAAGTTATAAT
TCGAAAAGGAGAATTGTTATGTGGAGTTCTAGATAAAACTCATTATGGTGCCACACCTTATGGCTTAGTTCATTGCA
TGTATGAATTGTATGGAGGTGATAGCTCTAGTGCACTTCTAAGTTCTTTCTCCAAAGTATTCACATTTTATCTACAA
TGGATAGGTTTTACTCTGGGAGTTAAAGATATTCTGGTTGTTGATGAAGCAAATAAACAAAGAGACAATTTTATCGG
CTTAGTAAGAGCAATTGGAAAAGTTGCTGCTGCAAAAGCCACCGATCTACCTGTTGATGTTGATGAAGTAAAGTTGA
AGGAAACTATAGGTGAAATGCTTACCAAGGATCCTAAATTCAGAGCTAATCTGGACAGGCAATACAAGAGTGTTTTA
GATTCATATACAAATAATATTAATACTGTATGCCTATCAGAAGGTTTGCTTGAGAAATTTCCTTATAATAATCTGCA
ACTGATGGTACAGTCTGGTGCAAAAGGATCAACTGTAAACACTATGCAAATTTCCTGTTTACTTGGCCAAATTGAGT
TGGAAGGTAAAAGACCGCCACTAATGATATCTGGAAGGTCTCTGCCTAGTTTTCCACCATACGATATATCCCCTAGA
GCAGGAGGTTTCATTGATGGACGTTTTATGACAGGCATTCAACCACAAGAGTTCTTCTTTCATTGTATGGCAGGTCG
TGAGGGTCTTATTGATACAGCTGTCAAAACAAGTCGTTCAGGTTATTTACAGCGATGTCTTATTAAACATTTAGAAG
GGCTAAGTGTCGCTTATGATCACACCGTGCGGGATTCTGATAGCAGTGTCATACAATTTGCGTATGGAGAAGACGGA
CTTGATGTATTAAAATGCCAATATTTTAATAAGAACCAATTCGAATTCCTTGATGTTAATTCAAACGCAGTCGTAAG

CAAATCCGTAATTAAGAAGTTAAAAGAAGATGATGAATCAAAGGCAATAGCAAAACTCCAAAAGTCATTGAAAAAAT
GGAAGAAGAAAAATGGATGTCCATTCCAAAAAGTGCGATATAGCCCATTCACAGAATTTTCTGCTATAGCTAAGAAT
GATATTGTCTTGGACGATAAACCAACAGATCAAACTAGAGACCCTAACTACTGGGAATTAGAAAAGATGTGGCGTAA
TTTAAGTGAAGATGAAAGACAAGAATACGCAAGAAAGCGCTGTCCAGATCCGATTCCAAGTAAATATTCACCAGAGT
ATAAATTTGGTGTTATAAACGAGCAATTGAATGAGCTTACACAAACATACTTGAAAAAACGAAAGGAGGACGCATAC
AGTGAATATACGGGGGAAAATAAGTTCACAGAGATAATAAATGCTAAGTATTTAGCTTCTATGGCAGCGCCGGGTGA
ACCGGTTGGATTGTTAGCAGCTCAATCTATTGGTGAACCCTCTACTCAGATGACACTGAACACTTTCCACTTTGCCG
GTCGGGGTGACATGAACGTCACCTTGGGTATTCCACGTCTTCGAGAAATTCTTATGACGGCTTCAGCGAAACTAAAA
ACTCCAAGCATGGATATTCCATTTCGAAGTGATTTGGCCGACTTAAACAAGAAGGCTGAGCGTCTCAGGCAAAAAAT
GAACAGAGTAACAGTATCAGACGTTCTTGAAAAAATTGACGTGCATTGCGAGATAGTCACCAATCCCAATAGGCAGC
TTAAAACCGTAATGCGCTTTTCATTTTTGCCTCATACTCAGTATAAAACGCAGTACGCGGTGAAACCACCACAGATT
ATAAAGCATATGCAGAACAAATTCTTCAGTGAAATGTTCTCCGTAATTCGTAAACAGGCTAAAACTACTTGTGGTGT
GATGTGGTCGACTGAGAAGGAGAAAAAGCGACGCGCAGCGACTGATGATGAGGATGAAGACGGAGAAGGCGCTTCTC
CAGATGTCGCTGAAAAAGCTGTTAATATGGATGGAGACAGTTCAGACGAAGATGGCCCGAACGACGATGACGATGAT
ACTGAT

>aly4523.3.2:T143C | DNA topoisomerase 3-beta-1
TCTACCGCAGCGTGTTCAGTCCATGAATGGAATGGCACATTCAAAGGCGAGTCAGTACGTTTTAAGATGACCTCTGT
GTGTGGTCATGTCATGAACTTGGATTTCACTGGTAAATACAACAACTGGGACAAAGTGGATCCCGTGGAGCTCTTCA
GTTGTCCCACGGAGAAGAAGGAAGCCATGCCCCGACTTCGGATACCGGCACATTTGGCTCAAGAGTCCCGCGGCGCT
GATTACTTGATTCTGTGGCTGGATTGCGATAAGGAAGGAGAAAATATTTGCTTTGAA

>aly499.37.1:G77G (not A) | Oocyte zinc finger protein XlCOF28 (Fragment)
ATGGAGGAAAACAAAAACATTACAAGAATAGATTTGACATCGTGTCGTATATGTTTGACCAACAAGAAACCATTGTG
TCCACTCTTTAAATATAAATTGTCTGGCAATTACGCTGAAATGTTGACTGCAATTGCCGACGTGAAG

>aly363.14.5:A76A (not C) | tRNA pseudouridine synthase-like 1
ACTAAAAATTTTAATATAGAAAAATTCAAAGAAGGCGCTAAGCATTTCTTAGGCTACCACGATTTCACGACTTTTAA
GAAGTTCGATAAATTGCAACAGAACAAGCAGAACCGTCGGACAATCTACTGCATAGACGTGCGGCCTGGCCGGCCCA
TGCTGTCCAGCTACTCAGAGAATAAACAGGACAGTCTGTTTGATTACTGGGACATAGAAATCAAGGCGAGGTCCTTT
GTGCATAATCAG

>aly2700.1.4:T70T (not G) | E3 ubiquitin-protein ligase RNF19A
TTCGCGGTGGTCGCGAGCGGTTGCGCCTCGTGCCCGAAGCTGACGTGCCTGGCGCCGGGCTGCGGGGCGTCGTTCTG
CTACCACTGCAAGGCGGCGTGGCACCCGACGCAGACGTGCGACGCCGCGCGCCGCAGCCGCCAGCCGCCGCCGCGCG
CGCCCGTCACCAGCCACAGCGAGATGGAGCAC

Barcinae:
>aly525.83.3:A682T | Glucose dehydrogenase [FAD, qui]
TTAGCGAACCCCGTATCGTCCTTCATGCAGTTCCTCCAAGAAGGCACAAGACAGCTCGATAACGAACCACCTGATCA
ATCGAGTATGCTCTCCGAATACGACTTCATTATAGTAGGTGCAGGTACGGCTGGCTGTGTTCTCGCCAATCGACTAT
CCGAGATTCCCGAATGGAAAATATTATTAATCGAAGCCGGAGTTAACGAAAACTTTGTCATGGATATACCTATCCTC
GCTAATTACCTGCAGTTCACACCGGCTAATTGGAGATACAAAACGAAACCGTCCGATAAATATTGCGCTGGATTCGA
AAATCAACAGTGTAATTGGCCCAGAGGTAAGGTTGTTGGAGGCTCAAGTGTTCTTAATTACATGATATACACACGAG
GAGCTCCTGCAGATTACGACAATTGGAAGGAAATGGGAAATGATGGTTGGGGATGGGACAACATATTGCCATACTTC
AAAAAAATTGAAAACTTCAATATTCCAGCTTTTGACGATCCTAAATATCATGGGCACGATGGACATCTGAACGTTGA
ACATGCCCCATTTCGCACCACAAAGGGAAAGGCTTGGGTGAAGGCAGCCCAAGAGTTAGGATTTAAGTATGGTGATC
ATAATGGAGCAAGCCCTTCTGGTATCTCGTTCTTACAGTTGTCTATGAAAAACGGCACGCGTCATAGTGCCAGCAGG
GCATACCTCCATCCCATTAACAGACGAAATAATCTACATTTAACAAAAATCAGTATGGTCACTAAATTACTTTTTGA
CCAAACAAAAACTAAAGTGATTGGGGTAGAATTAGAGAAACAAGGACGTAAACATAAGATATTAGCAAAGAAAGAAG
TCATAGTTTCTGCTGGTGCCATTAATTCCCCACAATTGCTCATGCTATCCGGTATTGGACCTAAAAGTCACTTGGAC
GAAATGAGTATACCTGTTGTCAAAGATTTACCTGTTGGCTACAATCTGATGGACCACATCGCTGCTGGTGGAGTACA
GTTTATGGTTAAAAGACAAAACTTTTCCCTA

>aly525.83.3:G683C | Glucose dehydrogenase [FAD, qui]

```
TTAGCGAACCCCGTATCGTCCTTCATGCAGTTCCTCCAAGAAGGCACAAGACAGCTCGATAACGAACCACCTGATCA
ATCGAGTATGCTCTCCGAATACGACTTCATTATAGTAGGTGCAGGTACGGCTGGCTGTGTTCTCGCCAATCGACTAT
CCGAGATTCCCGAATGGAAAATATTATTAATCGAAGCCGGAGTTAACGAAAACTTTGTCATGGATATACCTATCCTC
GCTAATTACCTGCAGTTCACACCGGCTAATTGGAGATACAAAACGAAACCGTCCGATAAATATTGCGCTGGATTCGA
AAATCAACAGTGTAATTGGCCCAGAGGTAAGGTTGTTGGAGGCTCAAGTGTTCTTAATTACATGATATACACACGAG
GAGCTCCTGCAGATTACGACAATTGGAAGGAAATGGGAAATGATGGTTGGGGATGGGACAACATATTGCCATACTTC
AAAAAAATTGAAAACTTCAATATTCCAGCTTTTGACGATCCTAAATATCATGGGCACGATGGACATCTGAACGTTGA
ACATGCCCCATTTCGCACCACAAAGGGAAAGGCTTGGGTGAAGGCAGCCCAAGAGTTAGGATTTAAGTATGGTGATC
ATAATGGAGCAAGCCCTTCTGGTATCTCGTTCTTACAGTTGTCTATGAAAAACGGCACGCGTCATAGTGCCAGCAGG
GCATACCTCCATCCCATTAACAGACGAAATAATCTACATTTAACAAAAATCAGTATGGTCACTAAATTACTTTTTGA
CCAAACAAAAACTAAAGTGATTGGGGTAGAATTAGAGAAACAAGGACGTAAACATAAGATATTAGCAAAGAAAGAAG
TCATAGTTTCTGCTGGTGCCATTAATTCCCCACAATTGCTCATGCTATCCGGTATTGGACCTAAAAGTCACTTGGAC
GAAATGAGTATACCTGTTGTCAAAGATTTACCTGTTGGCTACAATCTGATGGACCACATCGCTGCTGGTGGAGTACA
GTTTATGGTTAAAAGACAAAACTTTTCCCTA

>aly1139.27.4:G112T | E3 ubiquitin-protein ligase RNF168
AGCCCAGCTTTGATTGATGAAAACACCACCAGTTCTGACAGTTCTGCTTCTTCTAAAGTAGCTTTAGTCAAATTTAA
CAATCGGCATTCACCTGAAATTACTCCTACATATGGAAAGTTGCTTAAAAATTTTCTAGACAAAAAAATGAAGAGTG
GAACTGGGATTTGGAATAAAGAAAATGGAAAGAGTATAAATACAAAG

>aly1139.27.4:G113C | E3 ubiquitin-protein ligase RNF168
AGCCCAGCTTTGATTGATGAAAACACCACCAGTTCTGACAGTTCTGCTTCTTCTAAAGTAGCTTTAGTCAAATTTAA
CAATCGGCATTCACCTGAAATTACTCCTACATATGGAAAGTTGCTTAAAAATTTTCTAGACAAAAAAATGAAGAGTG
GAACTGGGATTTGGAATAAAGAAAATGGAAAGAGTATAAATACAAAG

>aly23605.15.15:G49A | CCR4-NOT transcription complex subunit 4
GACAACCTGGAGCGAGTCCGGCGCGCCCGGCTGGCGCCGCCCGGCTTCGGCCACGTCAGCGCGTTCGGCGGGCCCGC
GCCGCGCCACCAGACACACCACCAA


>COI barcode reference sequence, many positions are used as characters
AACTTTATATTTTATTTTTGGAATTTGAGCAGGATTAATTGGAACTTCATTAAGTTTACTTATTCGAACTGAATTAG
GAACTCCAGGATCTTTAATTGGAGATGATCAAATTTATAATACTATTGTTACAGCTCATGCTTTTATTATAATTTTT
TTTATAGTTATACCTATTATAATTGGGGGATTTGGAAATTGACTAGTACCCCTTATATTAGGAGCCCCAGATATAGC
TTTTCCTCGTATAAATAATATAAGATTTTGATTATTACCCCCATCTCTAACTCTTTTAATTTCAAGAAGTATTGTTG
AAAATGGAGCAGGTACTGGATGAACTGTTTATCCACCTTTATCTTCTAATATTGCCCATCAAGGAGCTTCAGTAGAC
TTAGCAATTTTTTCATTACATCTTGCAGGAATTTCATCTATTTTAGGAGCTATTAATTTTATTACAACTATTATTAA
TATACGAATTAATAATTTATCTTTTGATCAAATACCATTATTTATTTGAGCCGTTGGAATTACAGCTTTATTATTAT
TACTTTCATTACCTGTTTTAGCTGGAGCTATTACTATATTATTAACTGATCGAAATTTAAATACTTCATTTTTTGAT
CCTGCAGGTGGAGGAGATCCTATCTTATATCAACATTTATTT
```
